# Dissecting positive selection events and immunological drives during the evolution of adeno-associated virus lineages

**DOI:** 10.1371/journal.ppat.1012260

**Published:** 2024-06-17

**Authors:** Lirong Li, Runkuan Qin, Yunbo Liu, Yu-Shan Tseng, Weihan Zhang, Lin Yu, Mario Mietzsch, Xinkai Zou, Haizhou Liu, Guangwen Lu, Hongbo Hu, Robert Mckenna, Jinliang Yang, Yuquan Wei, Mavis Agbandje-Mckenna, Jiankun Hu, Lin Yang

**Affiliations:** 1 Department of Cardiology and Laboratory of Gene Therapy for Heart Diseases, State Key Laboratory of Biotherapy, and Collaborative Innovation Center for Biotherapy, West China Hospital, Sichuan University, Chengdu, Sichuan, China; 2 Department of Biochemistry and Molecular Biology, Center for Structural Biology, The McKnight Brain Institute, University of Florida, Gainesville, Florida, United States of America; 3 General Surgery Department, Gastric Cancer Center and Laboratory of Gastric Cancer, State Key Laboratory of Biotherapy and Collaborative Innovation Center of Biotherapy, West China Hospital, Sichuan University, Chengdu, Sichuan, China; 4 State Key Laboratory of Biotherapy and Cancer Center, Collaborative Innovation Center for Biotherapy, West China Hospital, Sichuan University, Chengdu, Sichuan, China; 5 Center for Immunology and Hematology, Department of Biotherapy, Cancer Center and State Key Laboratory of Biotherapy, West China Hospital, Sichuan University, Chengdu, Sichuan, China; 6 Computational Virology Group, Center for Bacteria and Viruses Resources and Bioinformation, Wuhan Institute of Virology, Chinese Academy of Sciences, Wuhan, Hubei, China; 7 Department of Emergency Medicine, State Key Laboratory of Biotherapy, West China Hospital, Sichuan University, Chengdu, Sichuan, China; 8 Laboratory of Aging Research and Cancer Drug Target, State Key Laboratory of Biotherapy and Cancer Center, National Clinical Research Center for Geriatrics, West China Hospital, Sichuan University, Chengdu, Sichuan, China; University of Wisconsin-Madison, UNITED STATES

## Abstract

Adeno-associated virus (AAV) serotypes from primates are being developed and clinically used as vectors for human gene therapy. However, the evolutionary mechanism of AAV variants is far from being understood, except that genetic recombination plays an important role. Furthermore, little is known about the interaction between AAV and its natural hosts, human and nonhuman primates. In this study, natural AAV capsid genes were subjected to systemic evolutionary analysis with a focus on selection drives during the diversification of AAV lineages. A number of positively selected sites were identified from these AAV lineages with functional relevance implied by their localization on the AAV structures. The selection drives of the two AAV2 capsid sites were further investigated in a series of biological experiments. These observations did not support the evolution of the site 410 of the AAV2 capsid driven by selection pressure from the human CD4^+^ T-cell response. However, positive selection on site 548 of the AAV2 capsid was directly related to host humoral immunity because of the profound effects of mutations at this site on the immune evasion of AAV variants from human neutralizing antibodies at both the individual and population levels. Overall, this work provides a novel interpretation of the genetic diversity and evolution of AAV lineages in their natural hosts, which may contribute to their further engineering and application in human gene therapy.

## Author summary

Adeno-associated virus (AAV) is a viral vector that is universally applied in human gene therapy. Despite their versatile applications in gene therapy, the origin and evolution of AAV serotypes have not been extensively studied. In this study, positive selection upon diversification of AAV capsid genes was carefully investigated by evolutionary analysis. Numerous diversifying selected sites were dispersed among the AAV lineages, and evolutionary pathways were inferred for two selected sites of the AAV2 capsid gene. Furthermore, the putative immunological drives underlying the selection of AAV2 capsid genes were experimentally dissected. This work provides a novel interpretation of the evolution and diversity of AAV serotypes and might be helpful for their engineering and application in gene therapy.

## Introduction

Adeno-associated viruses (AAVs) are small T = 1 viruses that package linear ssDNA and belong to the *Parvoviridae* family. As AAVs have the ability to transduce dividing and non-dividing cells, they have shown great promise as vectors for gene therapy [1]. To date, seven AAV biologics, such as Luxturna (2017) and Zolgensma (2019), have been approved by the U.S. Food and Drug Administration (FDA) and/or the European Medicines Agency (EMA) for the treatment of inherited diseases, and more than one hundred clinical trials are ongoing [2].

The isolation and characterization of AAV serotypes are important for understanding of the evolution of AAV and the development of gene therapy [3]. Currently, more than 100 AAV variants have been isolated from nonhuman primates and humans [4–6]. Historically, these viruses have been classified into 13 AAV serotypes with different tissue tropisms and immunogenicity. The AAV serotypes constitute an invaluable vector toolkit for human gene therapy because of their diverse biological properties. For instance, AAV9 was successfully applied to gene therapy for spinal muscular atrophy because of its superior transduction of motor neurons [7]. However, little is known about the evolutionary mechanism of these virus variants, except that a high rate of homologous recombination was reported among AAV capsid genes from a rhesus macaque with functional analysis inferring their variable serologic reactivity [4], which was also employed as an approach to genetically manipulate the recombinant AAV to expand its packaging capacity [8,9]. In contrast, the directed evolution approach based on Darwin’s natural selection theory has been utilized for genetic engineering of AAV capsid genes to improve their performance in gene therapy [10,11].

Compared with those of viral pathogens such as human immunodeficiency virus type 1 (HIV-1) and hepatitis C virus (HCV) [12,13], little is known about the interactions of AAV with human and nonhuman primates. Previous research on AAV has focused mainly characterizing its tissue tropism in rodent models, which was largely driven by the desire to develop it as a gene therapy vector [14]. Humoral immunity against AAV was subsequently emphasized in clinical trials because pre-existing AAV neutralizing antibodies inhibited vector transduction and therapeutic efficacy [15]. However, research on the interaction of AAV with its natural hosts has been largely ignored because of its relative non-pathogenicity and lack of significant impact on AAV-based molecular therapeutics. Investigations of the co-evolution of AAV and the human immune system may reveal patterns of interaction between AAV and human neutralizing antibodies, which could influence the application of the AAV vector in clinical trials of gene therapy. Furthermore, the potentially variable interactions of different AAV serotypes with the identified common cellular receptor AAVR might be an interesting topic of the important clinical significance [16].

In this study, we attempted to explore the evolutionary mechanism of AAV lineages in humans and nonhuman primates. Specifically, numerous positively selected sites with a sporadic distribution among different AAV capsid gene lineages were identified via the branch-site test [17] or mixed effects model of evolution (MEME) [18]. Furthermore, the evolutionary drive of the selection sites on the AAV2 capsid was further explored by mutagenesis and immunological experiments in animal models and human blood samples. While no evidence supported the interaction of the AAV2 capsid 410 site with host cellular immunity, replacement of the 548 site was correlated with immune evasion from human neutralizing antibodies. This work provides insights into the evolution and origin of AAV serotypes, which might contribute to the directed evolution and clinical application of this increasingly important gene therapy vector.

## Results

### Detection of positive selection in AAV evolution by codon-based models

The complete coding sequences of the 129 primate AAV capsid genes from GenBank were used, excluding those featuring artificial modifications or large deletions (S1 Table). The sequences were aligned using the codon-based PRANK algorithm to generate a sequence alignment for sitewise detection of positive selection [19,20]. Subsequently, a maximum-likelihood tree was generated from this sequence alignment using the DNAML program [21].

The M2a and M8 models in the CODEML program were initially used [22] to detect sites subject to positive selection across all of the AAV branches. No sites within AAV capsid genes were subjected to positive selection by the M2a model. However, two sites were inferred to be positively selected across AAV capsid gene variants by the M8 model, corresponding to the AAV2 residues T410 and S721 (Table 1).

**Table 1:** Detection of sites subject to positive selection through AAV capsid genes.

Evolutionary model in PAML software	Results without consideration of recombination	Results with consideration of recombination
M2a	No positive selection	No positive selection
M8	T410 ^*a*^, S721 ^*b*^	No positive selection

^*a*^ The probability for these sites subject to positive selection was larger than 95% by Bayes empirical Bayes analysis. The dn/ds ratio was larger than 1 for this site through the AAV lineages, but only sites correspondent in the AAV2 capsid were shown here.

^*b*^ The selected site on the exterior surface of AAV capsids is underlined.

However, as homologous recombination has been shown to occur during the evolution of AAV capsid genes [4,23], this could result in the erroneous detection of positive selection [24]. Hence, a strategy to first identify the recombination breakpoints within sequences was adopted, followed by the use of split sequence alignments without recombination for the detection of positive selection [25]. Three programs, GENECONV, Maxchi [26] and GARD, [27] were jointly implemented for the detection of recombination among AAV capsid genes. This analysis resulted in the detection of multiple recombination breakpoints; therefore, the AAV sequence alignment was accordingly partitioned into 23 segments longer than 3 codons. A maximum-likelihood tree was generated from each of these segments using the DNAML program [21]. The segments and their corresponding phylogenetic trees were then subjected to CODEML analysis using the M2a or M8 models. Intriguingly, no positive selection was detected by these models. The failure to detect positive selection by the M8 model could have resulted from the false-positive signal from the full-length capsid sequences without consideration of recombination or the lower power of CODEML for split sequence segments [28].

### Dissection of sporadic positive selection events among AAV lineages

The occurrence of positive selection tends to be sporadic on special branches or gene lineages, while this effect is often concealed by more prevalent neutral or purifying selection signals. Hence, the branch-site test of the CODEML program for the detection of episodic diversifying selection, which has integrates the variable selection pressure among branches into sitewise selection analysis, was used. Five AAV lineages correspondent to the AAV2, AAV1/AAV6, AAV5, AAV8 and AAV9 serotypes were selected for this analysis because of their distinct biological properties and important potential in gene therapy (Table 2). No sites within the AAV2 lineage were subjected to positive selection according to the branch-site test. Five sites, R92, S526, M599, V699 and P735 were subjected to positive selection within the AAV1/AAV6 lineage. Furthermore, the N642 site was uniquely selected for the AAV1 capsid gene branch. Residue R92 was subjected to positive selection for the AAV8 lineage according to a branch-site test. Two residues, Y478 and I479, were subjected to positive selection in the AAV9 lineage. Notably, R92 appeared to be the only selected site shared by multiple AAV lineages, while all the other selection events occurred alone.

**Table 2:** Detection of episodic diversifying selection among capsid genes of AAV lineages.

AAV lineages	Methods for selection analysis	
	Branch site test ^*a*^	MEME ^*b*^
AAV2	No selected sites	T410 ^*c*^, $\underline{E548}$ ^*d*^
AAV1/AAV6	R92, S526, M599, N642 ^*e*^, V699, P735	$\underline{T326}$, T411, K493, D495
AAV5	Too many selected sites ^*f*^	G15, R710
AAV8	R92	Q413, $\underline{A551}$, Q594
AAV9	Y478, I479	A273, $\underline{D327}$, Q412, N598

NOTE−All the selected sites displayed here were relevant to the emergence of one or more special AAV lineages. Furthermore, a number of serine codons were inferred subject to diversifying selection by the branch-site test or MEME within AAV lineages. However, the identical serine residue was observed for almost all the AAV lineages at these sites, which was thought to arise through the toggling selection or doublet mutation mechanism [29]. These sites were not included in the table because no observable replacement occurred over them.

^*a*^ The probability for these sites subject to positive selection was larger than 95% by Bayes empirical Bayes analysis.

^*b*^ These sites were shown with evidence of episodic diversifying selection at the significance level of 0.05 with the empirical Bayes factors of the identified branches not less than 20.

^*c*^ The selected sites were colored according to their evolutionary relationship, with the same color inferring that they were orthologous within the sequence alignment.

^*d*^ The selected sites on exterior surface of AAV capsids were underlined.

^*e*^ This selected site was detected for the AAV1 branch, while all the other five selected sites were shared by AAV1 and AAV6.

^*t*^Twenty-one selected sites were detected for the AAV5 branch by the branch-site test. As these results were suspicious due to the fact that the AAV5 capsid gene possessed the most sequence variations at the root of the phylogenetic tree in most conditions, they are not shown here.

Although the branch-site test was useful in the identification of positively selected sites, it required the branches to be tested for positive selection to be specified a priori. Therefore, another method, the mixed effects model of evolution (MEME), was employed to detect episodic diversifying selection during AAV evolution [18]. The unrecombined AAV sequence segments were sequentially subjected to MEME analysis. Intriguingly, positive selection was observed for almost all the AAV lineages (Table 2). Two sites, T410 and E548 were subjected to positive selection in the AAV2 lineage. Four sites, T326, T411, K493 and D495 were positively selected in the AAV1/AAV6 lineage. Two selected sites, G15 and R710, were identified in the AAV5 lineage. Three sites, Q413, A551 and Q594, were positively selected in the AAV8 lineage. Four sites, A273, D327, Q412 and N598, were subjected to positive selection in the AAV9 lineage. Interestingly, most of the selected sites identified by MEME analysis showed orthologous relationships in groups among the AAV lineages (with the same color indicating an orthologous group in Table 2), e.g., T410 in AAV2, T411 in AAV1 and AAV6, and Q413 in AAV8 or Q412 in AAV9, which inferred their functional conservation and common evolutionary pathways. More intriguingly, although no correspondence was observed between the results from the branch-site test and the MEME algorithm, defined correlations were observed between the selected sites from the M8 model and those from the MEME algorithm. In addition to the T410 site in AAV2 (orthologous to T411 in AAV1 and AAV6, Q413 in AAV8 or Q412 in AAV9), S721 in AAV2 (orthologous to the R710 site in AAV5) was simultaneously detected by the latter two computation methods (Tables 1 and 2).

**Fig 1:**
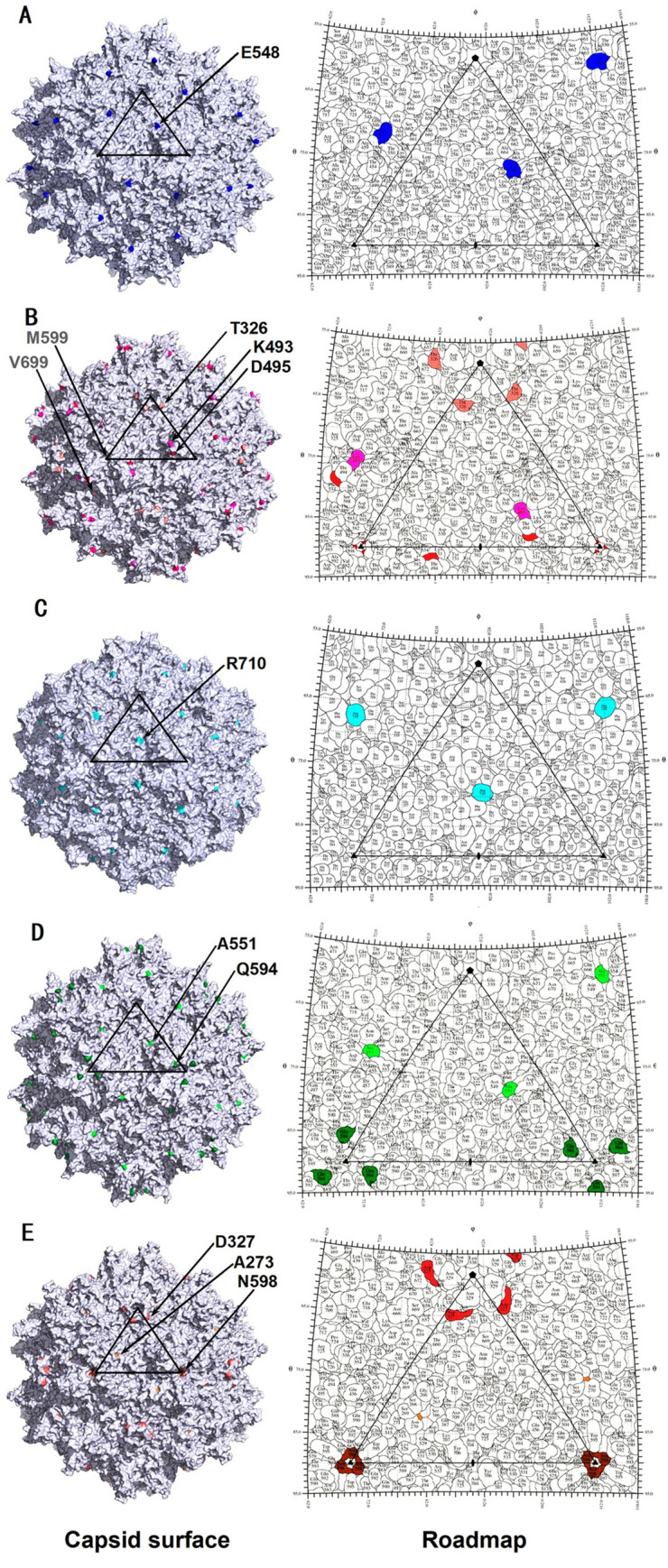
Distribution of positively selected sites of AAV lineages on their capsid structures. The positively selected residues for the AAV2, AAV1/6, AAV5, AAV8 and AAV9 lineages are highlighted on their respective capsid surface images (left side) and a stereographic roadmap projection (right side) in (A), (B), (C), (D) and (E), respectively. (A) AAV2, residue E548 (blue) was detected by the MEME method. (B) AAV1/6 lineage, residues M599 (red) and V699 (pink) (detected by the branch-site test) and T326 (salmon), K493 (light-magenta), and D495 (hot-pink) (detected by MEME) are displayed on the AAV6 capsid. (C) AAV5, residue R710 (cyan) was detected by MEME. (D) AAV8, residues A551 (light-green) and Q594 (forest-green) were both detected by MEME. (E) AAV9, residues A273 (orange), D327 (deep-salmon) and N598 (chocolate) were detected by MEME. The black triangle depicts a viral asymmetric unit, with the solid pentagon (5-fold), triangle (3-fold), and oval (2-fold) indicating the icosahedral symmetry axes respectively. In the stereographic Roadmap projections, the boundary for each residue is in black, and the residues are as labeled.

To further examine possible correlations with AAV biology, the positively selected sites were identified on the structures of the AAV serotypes (Fig 1). Of the two sites selected for the AAV2 lineage by MEME, E548 was observed around the threefold protrusion of the capsids. Of the five selected sites detected for the AAV1/AAV6 lineage by the branch-site test, M599 and V699 were observed on or around the threefold protrusion of the AAV6 capsids, respectively. Moreover, three sites inferred by MEME for this AAV lineage were on the external surface, with T326 around the fivefold plateau and K493 and D495 on the threefold protrusion. One of the two selected sites for the AAV5 lineage from MEME analysis, R710, was localized around the twofold canyon of the capsids. Two of the three selected sites detected by MEME for the AAV8 lineage, A551 and Q594, were found around or on the threefold protrusion, respectively. Furthermore, three of the four selected sites inferred by MEME were on the external surface of the AAV9 capsids, with D327 on the fivefold channel, A273 on the 2/5-fold wall and N598 on the threefold protrusion. Generally, most of the selected sites detected by MEME were on the exterior surface of AAV capsids, which strongly suggested their potential roles in tissue tropism and/or the immune response to AAVs.

The functional implications of the selected sites from the AAV lineages were further explored by referring to the related references (Table 3). The residue orthologous to the R92 site in the AAV1/AAV6 or AAV8 capsid was in the SH2 binding motif of the AAV2 VP1 N-terminus [30], and rapid evolution of its orthologous site in the AAV1, AAV6 and AAV8 capsid genes might alter their tissue tropism. The T410 site was identified in an epitope that elicits IFN-γ secretion by human PBMC in an ELISPOT assay [15]. Mutations of its orthologous sites in the AAV2, AAV1/AAV6, AAV8 and AAV9 lineages might correlate with immune evasion from T cells. The K493 site of the AAV6 capsid participated in its interaction with heparin, the evolution of which might correlate with the transduction property of the AAV1/AAV6 serotypes [31]. The K493 and D495 sites of AAV1 were within the footprint of the monoclonal antibody 4E4 [32], and replacements at these sites may affect the antibody recognition of the AAV1/AAV6 lineage. The E548 site of the AAV2 capsid was in the footprint of the A20 antibody [33], and mutation of its orthologous sites in the AAV2 and AAV8 lineages might be related to their evasion of neutralizing antibodies. The Q594 site of the AAV8 capsid was reported to interact with its receptor, the 37/67-kDa laminin receptor [34]. Furthermore, in related structural biological research, the Q594 site of AAV8 and the R710 site of AAV5 were shown to interact with the crucial AAV cellular receptor AAVR [35,36]. Thus, the evolution of these two sites might facilitate host adaptation of the AAV8 and AAV5 lineages.

**Table 3:** Putative functional implications of positively selected sites in AAV capsids.

AAV selected sites /serotypes	Potential functions	References
R92/AAV1, AAV6 and AAV8	Within the SH2 binding motif of the AAV2 VP1 N-terminus	[30]
T410/AAV2, T411/AAV1 and AAV6, Q413/AAV8 or Q412/AAV9	Within the T-cell epitope of peptide 82 on the AAV2 capsid	[15]
K493/AAV1 and AAV6	Participates in heparin binding of AAV6	[31]
K493 or D495 /AAV1 and AAV6	Within the footprint of monoclonal antibody 4E4 on the AAV1 capsid	[32]
E548/AAV2 or A551/AAV8	Within the footprint of monoclonal antibody A20 on the AAV2 capsid	[33]
Q594/AAV8	Within the interacting domain of AAV8 with its potential receptor LamR	[34]
Q594/AAV8 and R710/AAV5	Within the interface residues of AAV2 or AAV5 with the cellular receptor AAVR	[35,36]

NOTE−As indicated in Table 2, the colorized selected sites illustrate their orthologous relationship in the same color. The K493 and D495 sites are shared by AAV1 and AAV6, and both of them were detected in the footprint of monoclonal antibody 4E4 on the AAV1 capsid. They were thus deduced to be in its footprint on the AAV6 capsid. The interface residues of AAV capsids with AAVR were only revealed for AAV2, AAV1 and AAV5 [35,36]. In accordance, the interface domain of the AAV8 capsid and AAVR was modelled by superimposition of the AAV8 structure onto that of AAV2/AAVR.

### Phylogenetic reconstruction of positive selection events in AAV lineages

Ideally, sites of positive selection identified through bioinformatics analysis should be experimentally validated. Notably, most of the selected sites from the MEME analysis were located on the exterior surface of AAV capsids and have potential functional implications (Fig 1 and Table 3). Two of these selected sites in the AAV2 lineage were selected for further exploration because AAV2 is the most prevalent serotype in human populations [37] and has been universally applied in clinical gene therapy [2].

**Fig 2:**
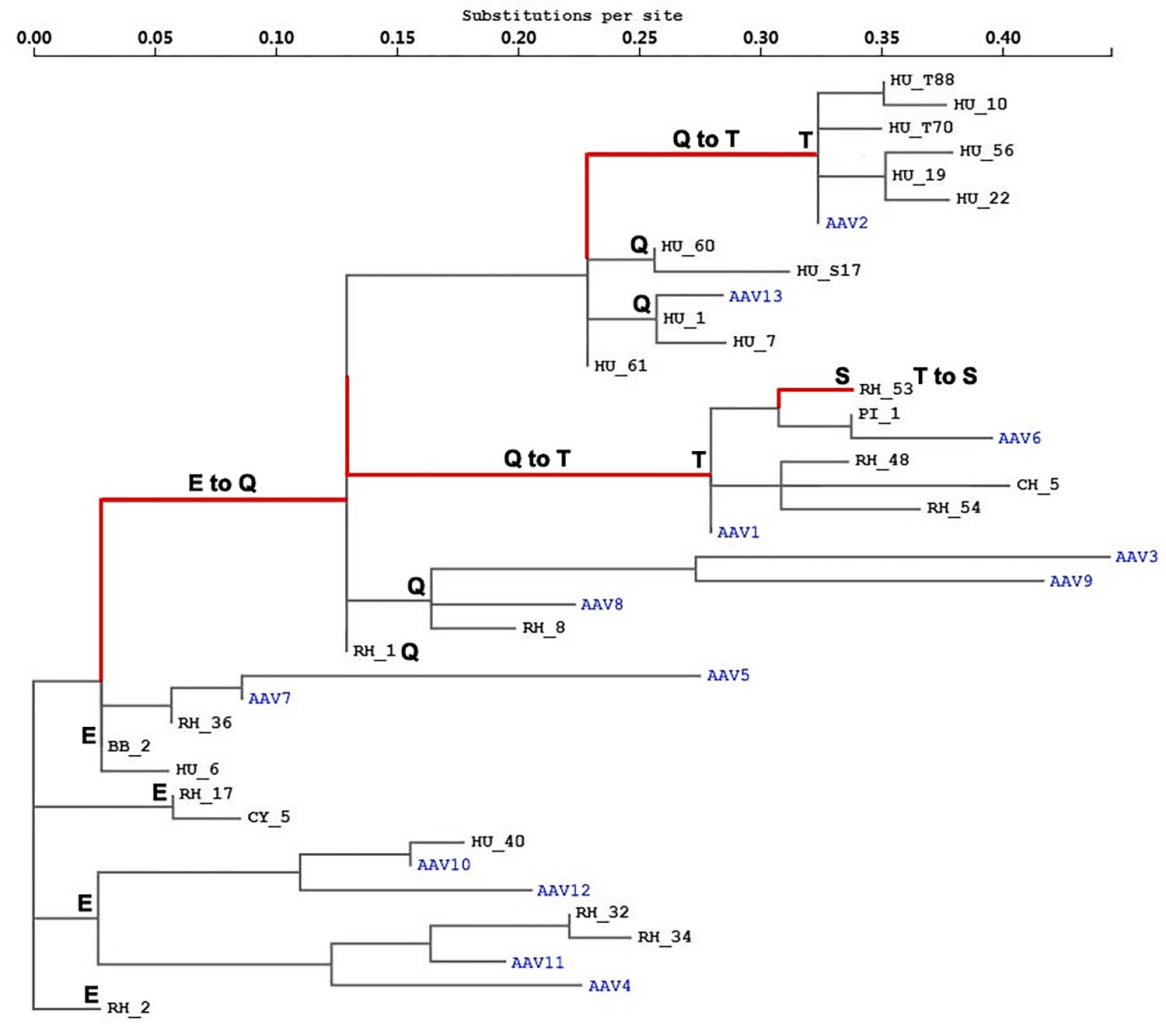
Selection analysis of AAV capsid coding sequences and the evolutionary pathway of AAV2 capsid site 410. The full-length AAV capsid coding sequences were subjected to recombination analysis and then partitioned into segments without recombination. The segment 1735–1773 was used for reconstruction of a phylogenetic tree with the global MG94 x REV model by the MEME program. The sequence isolates were labeled with reference to the source species (HU, human; RH, rhesus macaque; CY, cynomolgus macaque; BB, baboon; CH, chimpanzee) or further to the related human tissues (T, tonsil/adenoid; LG, lung; S, spleen) [5,6]. Partial AAV isolates were removed from the tree because of their duplicated sequences. Site 410 was identified as experiencing positive diversifying selection on a number of branches, which are shown in red. To further illustrate the evolutionary pathway of this site, the amino acid residues corresponding to each branch or cluster were annotated, and the replacement events relevant to positive selection are shown above or adjacent to the branches. The scale for genetic distance is indicated at the top.

**Fig 3:**
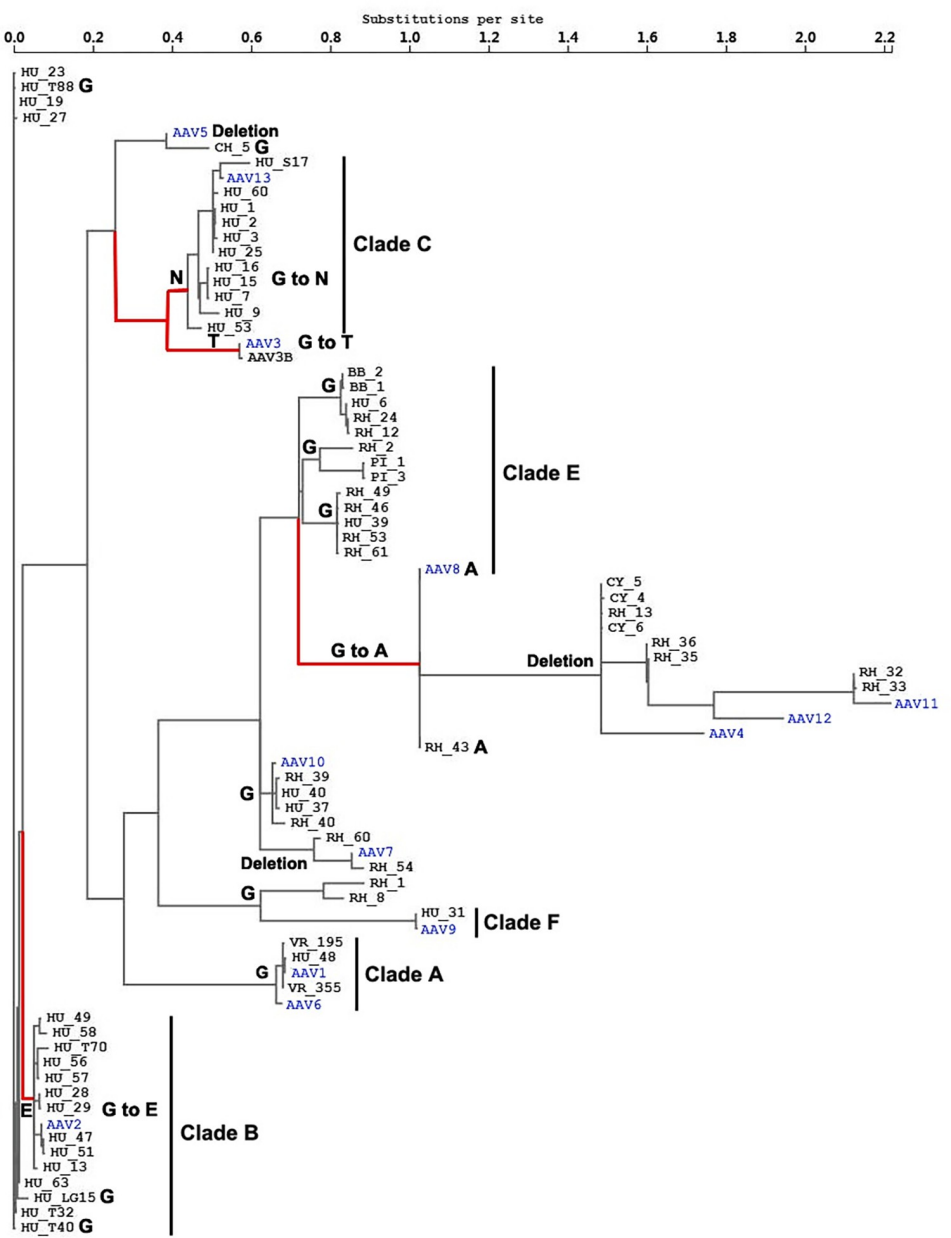
Selection analysis of AAV capsid coding sequences and the evolutionary pathway of AAV2 capsid site 548. After recombination analysis of the full-length AAV capsid coding sequences, segment 2572–3468 was used for phylogenetic reconstruction with the global MG94xREV model by the MEME program. The sequence labeling was the same as that in Fig 2, with the redundant sequences removed for clear observation. The major AAV clades are indicated to the right of the taxa, as previously described [5]. Site 548 was identified as experiencing positive diversifying selection on some branches, which are colored in red. To further illustrate the evolutionary pathway of this site, the amino acid residues corresponding to each branch or cluster were annotated, and the replacement events inferring positive selection are displayed below or adjacent to the branches. The scale for genetic distance is shown at the top.

A fundamental understanding of the evolutionary history of the two selected sites among AAV lineages might be needed (Table 2). A phylogenetic tree was first constructed based on the 1735–1773 segment of the AAV capsid coding sequence alignment to illustrate the selection pressure and replacement events on site 410 of the AAV2 capsid (Fig 2). The ancestral codon at this site in AAV4 and AAV5 lineages was glutamic acid. A cascade of positive selection events succeeded in occurring along the phylogenetic tree (marked in red in Fig 2), with the first substitution from glutamic acid to glutamine in the AAV8 and AAV9 lineages, the second replacement from glutamine to threonine in the AAV1/AAV6 and AAV2 lineages, respectively, and a more recent replacement from threonine to serine in the rh.53 branch. Similarly, the evolutionary history of site 548 of the AAV2 capsid was dissected from the 2572–3468 segment of the AAV capsid coding sequences based on a phylogenetic tree (Fig 3). The phylogenetic tree was rooted as glycine, which is shared by the AAV1/AAV6, AAV7 and AAV9 lineages. Diversifying selection was then observed to occur in parallel in four AAV lineages (marked in red in Fig 3), including glycine to glutamic acid in the AAV2 lineage, glycine to alanine in the AAV8 lineage, glycine to threonine in the AAV3/AAV3B lineage, and glycine to asparagine in the AAV13 lineage.

Hence, an attempt to address the effects of selection pressure on the evolution of AAV lineages with the AAV2 capsid gene was further pursued.

### Dissecting the immunological drive for the evolution of site 410 in the AAV2 capsid lineage

**Fig 4:**
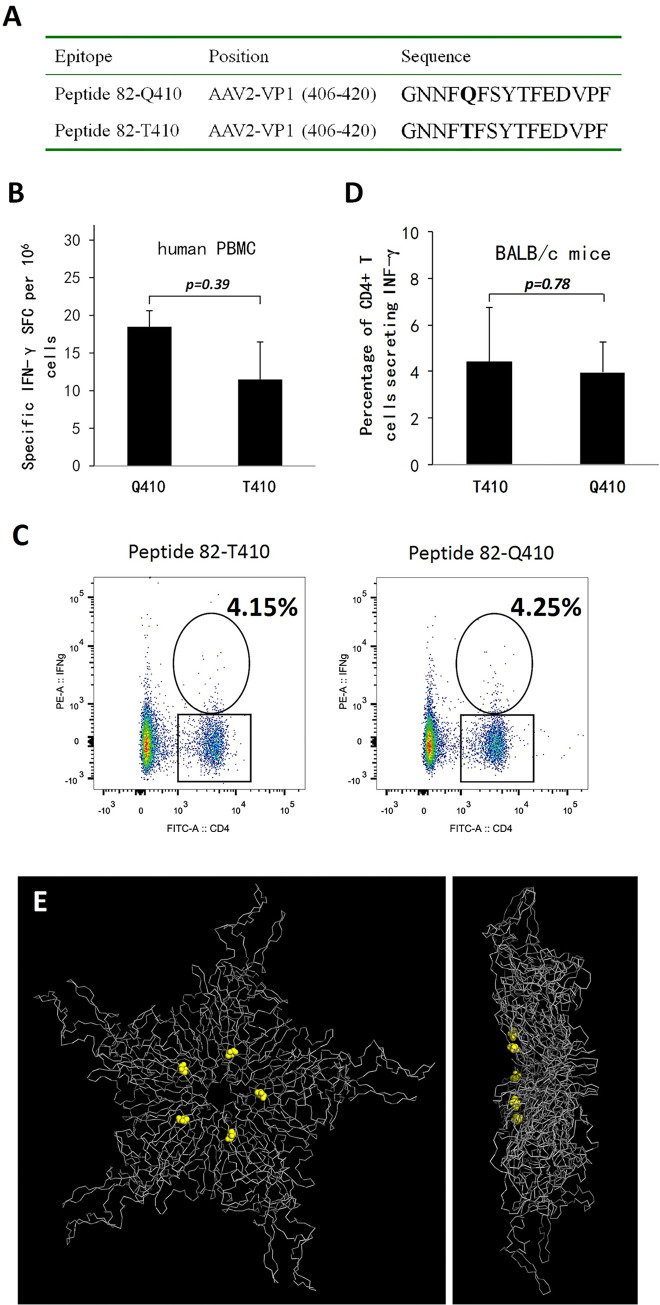
Exploration of the immunological drive for the evolution of site 410 of the AAV2 capsid gene lineage. (A) The sequence of the 15-mer peptide 82, with site 410 being variable among AAV lineages. This T-cell epitope was originally identified in a human subject by the IFN-γ ELISPOT assay [15]. Peptide 82, with glutamine (Q) and threonine (T) at site 410, was hypothesized to represent cognate or variant epitopes with variable affinities recognized by human or mouse MHCII molecules (S2 Table). (B) ELISPOT assay to examine the immunological effects of a mutation at site 410 from Q to T in the AAV2 capsid in a human subject. Approximately 100 human plasma samples were prescreened by ELISA for hu.1 capsid binding, which contains the peptide 82-Q410 epitope with a high prevalence in human populations [5]. PBMCs were then isolated from the positive blood samples to detect IFN-γ secretion by ELISPOT assay after incubation with peptide 82-Q410. Finally, human PBMC samples that were positive according to both ELISA and ELISPOT prescreening were subjected to IFN-γ ELISPOT assays to compare the mutation effects of the peptides 82-Q410 and 82-T410 using a paired t-test. (C), (D) Immunological effects of replacement at site 410 of the AAV2 capsid in BALB/c mice. An MHCII-binding peptide analogous to peptide 82 was predicted by RANKPEP analysis in BALB/c mice. Four BALB/c mice from each group were intramuscularly immunized with Ad-AAV2Cap or PBS. Splenocytes were harvested 9 days after immunization for incubation with the peptides 82-T410 or 82-Q410 before intracellular cytokine staining to detect IFN-γ secretion in CD4+ T cells. Representative flow cytometry images are shown in (C), and an unpaired t-test was performed to compare the reactivity of CD4^+^ T cells to AAV2 epitopes in (D). (E), (F) Localization of site 410 in the AAV2 structure. A ribbon model of an AAV2 VP3 pentamer is shown from the inside of the capsid (E) and in a side view (F). The 410 sites are represented as sphere models in yellow.

Although site 410 of the AAV2 capsid was not localized on its exterior surface, it was contained in peptide 82, which was originally identified by an ELISPOT assay of PBMCs from a human subject in a gene therapy clinical trial [15]. When the AAV2 VP1 sequence was subjected to RANKPEP analysis [38], multiple MHCII genotypes were predicted to recognize epitopes analogous to peptide 82, including HLA-DR2, HLA-DR4 (DRB1*0401), HLA-DR5 and HLA-DR8 (DRB1*0801) (S2 Table). A plausible deduction for this observation was that the evolution of site 410 in the AAV2 capsid was driven by selection pressure from human CD4+ T-cell recognition, with epitopes harnessing glutamine and threonine at site 410 assumed to be the cognate and variant peptides, respectively (Fig 4A), considering the evolutionary pathway illustrated in Fig 2.

The AAV variant hu.1 was immediately ancestral to AAV2 in the phylogenetic tree (Fig 2) and possessed the peptide 82-Q410 epitope (GenBank No. AY530575). Furthermore, clade C, represented by hu.1, was found to be highly prevalent in human populations in a previous report [5]. Based on these data, we deduced that human subjects with past hu.1 infection might be appropriate for exploring the selection mechanism for mutations within peptide 82. A gastric cancer cohort of 94 patients was recruited from West China Hospital for this study. In fact, 36 of these plasma samples tested positive for antibodies targeting the hu.1 capsid according to ELISA. Whole blood from these individuals was further used for the isolation of PBMCs and the detection of IFN-γ secretion by ELISPOT with peptide 82-Q410 as the immunogen. Three human PBMC samples demonstrated a positive T-cell response. However, reproducible results were obtained for only one of them when IFN-γ ELISPOT was used for comparison of their reactivity to the peptides 82-Q410 and 82-T410 (Fig 4B). The peptide 82-T410 moderately reduced the stimulatory reactivity of this human PBMC sample, but this difference was not statistically significant (p = 0.39, Fig 4B).

Interestingly, an epitope analogous to peptide 82 was also identified by RANKPEP [38] for the BALB/c mouse strain, which carried the MHCII I-Ad allele (S2 Table). This led to the idea that the mutation effects of the AAV2 capsid 410 site on T-cell immunity could also be examined in this mouse model. BALB/c mice were immunized with Ad-AAV2Cap, and splenocytes were tested 9 days later by intracellular cytokine staining for CD4+ T-cell responses to the peptides 82-T410 or -Q410 (Fig 4C and 4D). In this case, the primary immunogen, i.e., the wild-type AAV2 capsid, contained the peptide 82-T410. Mouse CD4+ T-cells showed only marginally reduced reactivity upon incubation with the peptide 82-Q410, but the difference was not statistically significance (p = 0.78, Fig 4D).

Overall, the current experimental data did not support a strong relationship between host cellular immunity and accelerated evolution of the AAV2 capsid 410 site. When the position of the T410 residue was visualized on the AAV2 capsid, this site was located surrounding the fivefold symmetric pore on the interior surface of the capsid (Fig 4E and 4F). As this channel is crucial for the release of the VP1 unique N-terminus before the endosomal escape of AAV [39], the T410 site is plausibly involved in the interaction with the VP1 N-terminus before exposure to the capsid surface, which warrants further investigation of the mutational effects of this site on viral tissue tropism in the future.

### Effects of mutations at site 548 of the AAV2 capsid on the mouse antibody response

Humoral immunity plays a crucial role in the defense against AAV infection in humans [15]. The accelerated evolution at site 548 of the AAV2 capsid gene might be driven by human neutralizing antibodies because it is a contact residue of the mouse monoclonal antibody A20 [33]. The A20 antibody was originally isolated from mice immunized with the wild-type AAV2 capsid, i.e., AAV2-E548 [40]. As a glycine residue was identified as the ancestral residue at site 548 during the evolution of the AAV2 lineage (Fig 3), examination of the neutralization effects on AAV2-G548 and AAV2-E548 using a monoclonal antibody against the AAV2-G548 capsid might help to test the hypothesis that rapid evolution of this site was driven by host humoral immunity.

**Fig 5:**
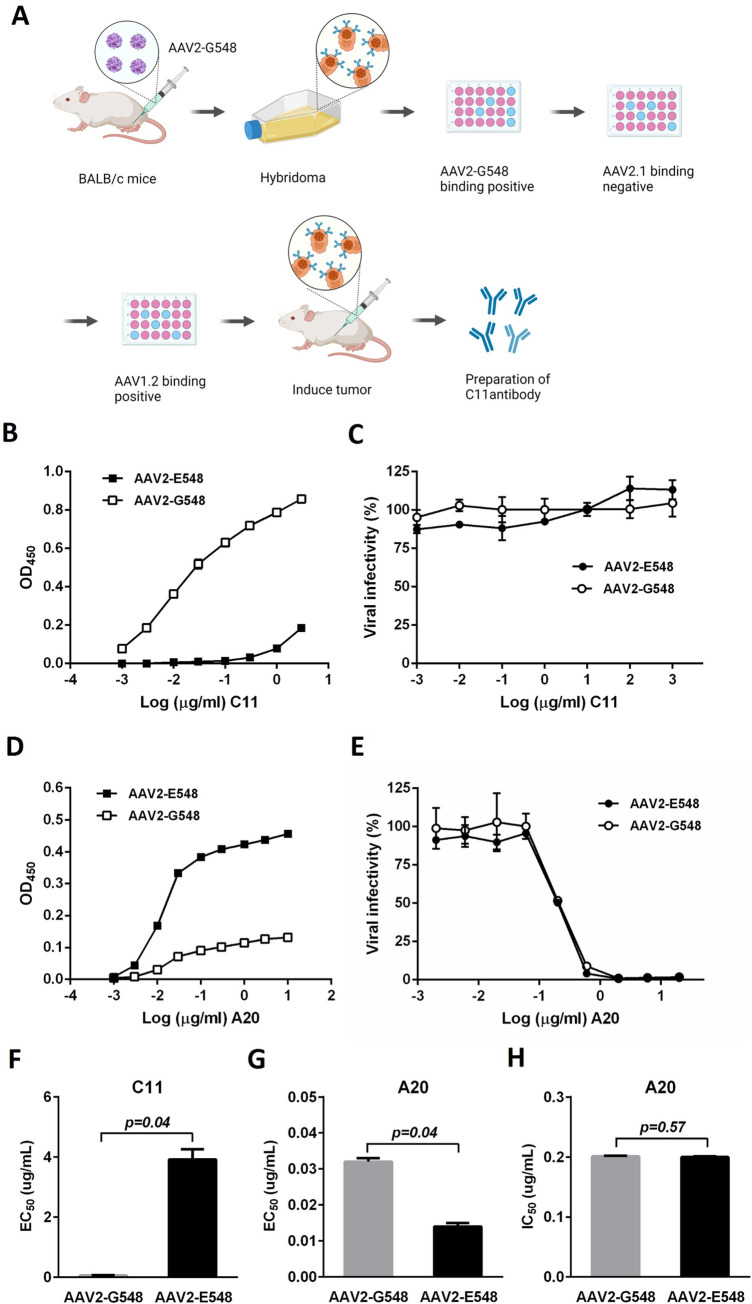
Mutational effects of AAV2 capsid site 548 on the binding and neutralization of mouse monoclonal antibodies. (A) Preparation of mouse C11 monoclonal antibody. The AAV2-G548 vector containing the lacZ transgene was used for intramuscular immunization of BALB/c mice, and splenocytes were subsequently isolated for fusion with the SP2/0 myeloma cell line. The generated hybridomas were diluted for culture and ELISAs using a panel of engineered AAV vectors, including AAV2-G548, AAV2.1 and AAV1.2, to screen the antibody with the cognate epitope with that of mouse A20 monoclonal antibody [33]. Created by BioRender.com. (B) Binding of the AAV2-G548 and AAV2-E548 capsids to a mouse C11 monoclonal antibody, as determined by by ELISA. (C) Neutralization of the AAV2-G548 and AAV2-E548 vectors by the C11 antibody. The recombinant AAVs harnessing the lacZ gene were incubated with a serially diluted antibody at 37°C for 1 hour before being inoculated into 293 cells. Transgene expression was monitored 24 hours later by a beta-galactosidase assay. (D) Binding of the AAV2-G548 and AAV2-E548 capsids with a mouse A20 monoclonal antibody. (E) Neutralization of the AAV2-G548 and AAV2-E548 vectors by the A20 antibody. The data in (B, D, E) were further used for calculation of EC_50_ values from ELISAs (F, G) and IC_50_ values from neutralization experiments (H) for comparison between the two AAV2 variants using paired t-tests. Each experiment was performed in duplicate, and the data are shown as the mean values ± SDs.

The neutralizing antibody appropriate for this purpose should ideally possess epitopes similar to those of the A20 antibody [33]. A comparison of the capsid sequences of the AAV serotypes revealed that AAV1 and AAV2 had greater sequence identity but with significant variations in the A20 epitopes, which accounted for their distinct antigenicity against this antibody (S1 Fig). This observation led to the design and construction of two AAV variants to screen antibodies against AAV2-G548. AAV2.1 is based on the AAV2 capsid sequence, with the seven residues in the A20 epitopes completely replaced with the corresponding residues from the AAV1 capsid. Negative screening using this AAV variant might eliminate antibodies against the AAV2 capsid with only those with epitopes similar to those of A20 retained. The AAV1.2 was constructed based on the AAV1 capsid sequence with six mutations introduced to ensure that the corresponding regions were identical to those in the AAV2/A20 epitopes. The only exception was that site 549 was retained as glycine, to be consistence with that in AAV2-G548, which is the antigen used for mouse immunization. Positive screening using this AAV variant would favor the selection of antibodies with a focus on the AAV2/A20 epitope. By performing iterated ELISA screenings with AAV2-G548, AAV2.1 and AAV1.2 after mouse immunization with AAV2-G548, ideal antibodies against AAV2-G548 with epitopes similar to those of AAV2/A20 could be obtained (Fig 5A).

Eventually, five monoclonal antibodies against the AAV2-G548 capsid were obtained following these procedures. When C11, a representative antibody, was used for the binding assays of AAV2-G548 and AAV2-E548, a high affinity was demonstrated for AAV2-G548 (Fig 5B). In contrast, binding to C11 was reduced 70-fold when AAV2-E548 was used for ELISA although only one replacement occurred between these two AAV2 variants (average EC_50_ = 0.056 and 3.922 μg/ml for AAV2-G548 and AAV2-E548, respectively, p = 0.04, Fig 5F). Furthermore, we noted that the OD_450_ value of AAV2-E548 was significantly lower at the highest C11 concentration (10 μg/ml) than that of AAV2-G548 (0.858 and 0.186 for AAV2-G548 and AAV2-E548, respectively, p = 0.02, Fig 5B). However, no neutralization of AAV2-G548 or AAV2-E548 was observed even when 1 μg/μl C11 was used for incubation with these viruses (Fig 5C). Furthermore, the other four monoclonal antibodies from the AAV2-G548 immunization were also verified to be non-neutralizing for AAV2 variants. These results showed a severe attenuation of affinity when a G to E mutation was introduced into the 548 site of the AAV2 capsid, but its effects on the neutralization properties of the antibodies could not be assayed.

As mentioned above, A20 is an extensively studied mouse monoclonal antibody capable of neutralizing the wild-type AAV2 capsid. As expected, AAV2-E548 showed efficient binding of the A20 antibody by ELISA, which contrasted with the lower affinity of AAV2-G548 (Fig 5D). Statistical significance was observed for the average EC_50_ values (0.032 and 0.014 μg/ml for AAV2-G548 and AAV2-E548, respectively, p = 0.04, Fig 5G) as well as for the OD_450_ readings at the highest A20 concentration (10 μg/ml) (0.132 and 0.457 for AAV2-G548 and AAV2-E548, respectively, p = 0.006, Fig 5D) between the two AAV2 variants. Intriguingly, the A20 antibody neutralized both of these AAV2 variants effectively (Fig 5E). Furthermore, no significant difference in neutralization potency was detected (average IC_50_ = 0.201 and 0.200 μg/ml for AAV2-G548 and AAV2-E548, respectively, p = 0.57, Fig 5H). These results suggested that the replacement of G with E at site 548 of the AAV2 capsid could significantly change its binding with the A20 antibody, but did not alter its antigenicity in neutralization experiments.

### Binding and neutralization of AAV2 variants by individual and pooled human plasmas

**Fig 6:**
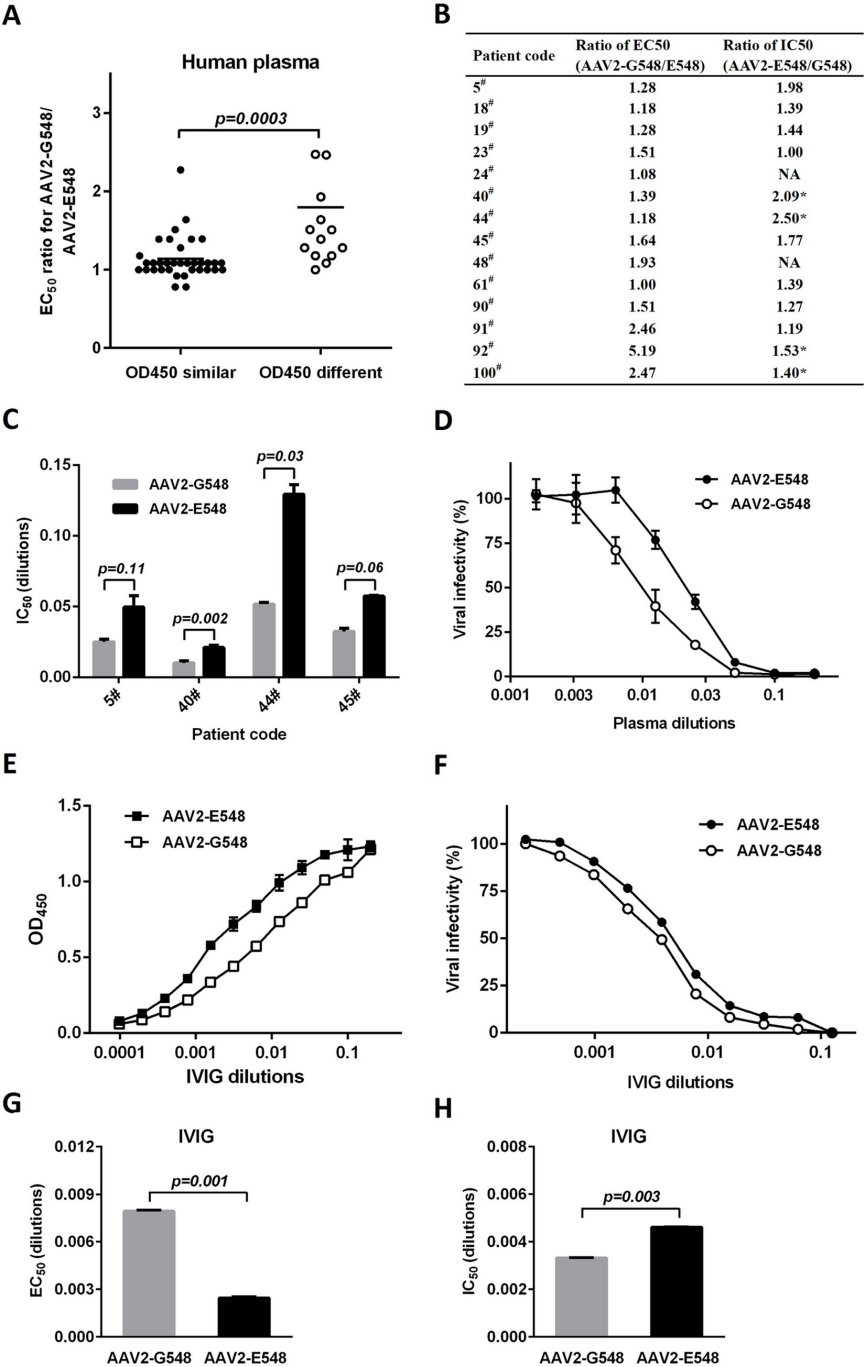
Immunological effects of AAV2 capsid mutations on the binding and neutralization of human antibodies. (A) Binding of the AAV2-E548 and AAV2-G548 capsids to individual human plasma samples by ELISA. The 49 human samples were roughly categorized into two groups based on their distinct OD450 readings in the preliminary experiment. The calculation and comparison of their EC_50_ values for AAV2-G548 and AAV2-E548 further supported the significant differences between these groups according to the Mann-Whitney test. (B, C, D) Neutralization experiments on the 14 human plasma samples selected by their different antibody titers against AAV2-G548 and AAV2-E548 (the different OD_450_ groups in (A)). Recombinant AAVs expressing the lacZ gene were incubated with serially diluted antibodies at 37°C for 1 hour before being inoculated into 293 cells. Transgene expression was monitored 24 hours later by a β-galactosidase assay. Two of the plasma samples did not neutralize AAV2 capsids (indicated by NA), and eleven of the remaining twelve plasma samples showed higher IC_50_ values for AAV2-E548 than for AAV2-G548 (B). Furthermore, the IC_50_ values from the four human plasma samples with greater differences between the two AAV2 variants are displayed in (C), and a representative neutralization curve is shown for plasma sample number 40# in (D). The indicated p values were obtained by paired t-tests in (C), with the significant difference between IC_50_ values at the 0.05 level shown by asterisks in (B). (E) Binding of the AAV2-E548 and AAV2-G548 capsids to pooled human plasma (IVIG) by ELISA. (F) Neutralization of the AAV2-E548 and AAV2-G548 vectors by IVIG. Paired t-tests were performed to compare the EC_50_ values of the AAV2-G548 and AAV2-E548 capsids for binding (G) and the IC_50_ values for neutralization (H) by IVIG. The data are shown as the mean values ± SDs.

It remains to be addressed whether the mutation at site 548 of the AAV2 capsid was driven by human humoral immunity. As 49 human plasma samples were collected during the course of the study to screen individuals potentially positive for a T-cell response to peptide 82, these samples could be used for ELISAs to examine the binding of AAV2 variants. Generally, AAV2-E548 showed stronger affinity for human antibodies than did AAV2-G548 in these experiments. The human plasma samples were roughly divided into two groups, with 35 individuals showing similar OD_450_ readings between the two AAV variants and the other 14 individuals showing much larger readings for AAV2-E548. The EC_50_ ratios were calculated and compared between two AAV variants for each plasma sample, and the results verified that this grouping was supported statistically, with the group of 14 individuals displaying significantly greater EC_50_ ratios against AAV2-G548 than against AAV2-E548 (p = 0.0003, Fig 6A and 6B).

These 14 human plasma samples were then further examined for their neutralization of the AAV2-G548 and AAV-E548 capsids. Interestingly, except for the two samples without neutralization effects (sample numbers 24# and 48#), the majority of these human plasma samples (eleven of twelve) showed more efficient neutralization of AAV2-G548 than of AAV2-E548, with four of them further exhibiting significant differences (p < 0.05, Fig 6B). The results of the four representative human plasma samples with the largest IC_50_ ratios were compared between the two AAV2 variants (Fig 6C). Furthermore, a typical neutralization curve was illustrated for human plasma sample number 40# (Fig 6D), which displayed the largest difference in the neutralization of AAV2-G548 and AAV2-E548 (average IC_50_ = 0.0102 and 0.0212 dilutions for AAV2-G548 and AAV2-E548, respectively, p = 0.002, Fig 6C).

Considering the variable phenotypes of AAV2 variants in the binding and neutralization of individual human plasma samples, this phenomenon was further investigated at the population level. Intravenous immunoglobulin (IVIG) derived from pooled human plasma from thousands of healthy donors was used for this study. Intriguingly, even pooled human plasma also showed a greater affinity for AAV2-E548 than for AAV2-G548 (average EC_50_ = 0.00794 and 0.00247 dilutions for AAV2-G548 and AAV2-E548, respectively, p = 0.001, Fig 6E and 6G). Furthermore, these human antibodies had a weaker neutralizing effect on AAV2-E548 than on AAV2-G548 (average IC_50_ = 0.00333 and 0.00462 dilutions for AAV2-G548 and AAV2-E548, respectively, p = 0.003, Fig 6F and 6H). All these results demonstrated a more universal prevalence but lower neutralization sensitivity of the AAV2-E548 capsid than of the AAV2-G548 capsid in human populations.

### Directed evolution and characterization of AAV2 capsid variants at site 548

The abovementioned data revealed the correlation between accelerated evolution of the AAV2 capsid site at position 548 and selection pressure from human neutralizing antibodies. An AAV2 library was then constructed by the introduction of saturation mutagenesis at the 548 site of its capsid gene. This library was administered to severe combined immune deficiency (SCID) mice infused with pooled human plasma (IVIG) to select AAV2 variants with potentially more efficient evasion of human neutralizing antibodies. The SCID-IVIG mouse model is considered an excellent animal model for mimicking human humoral immunity [41].

**Fig 7:**
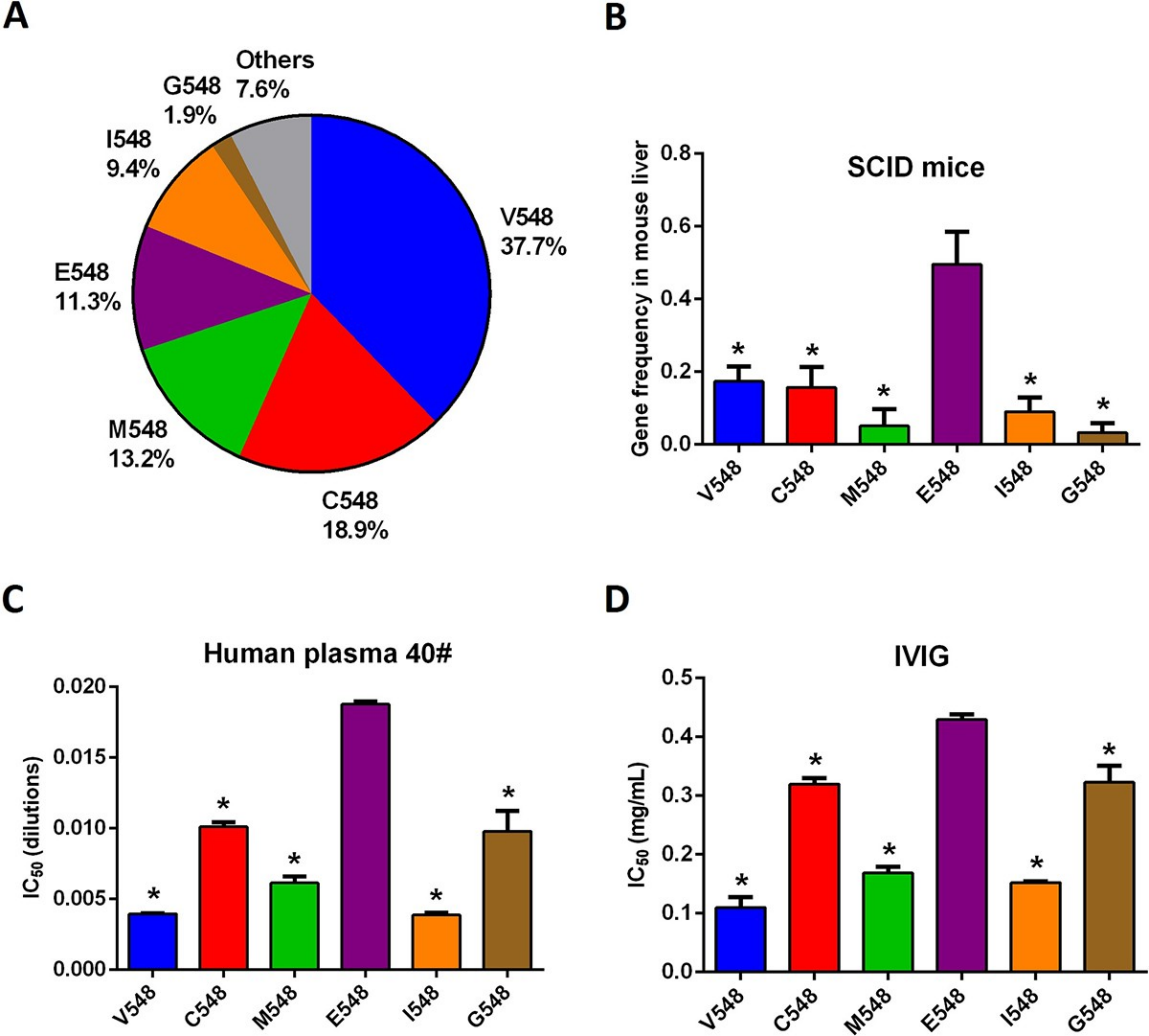
Evolution of AAV2 variants in a mouse model in the presence of human neutralizing antibodies. (A) Distribution of AAV2 variants in mouse liver after one round of screening. An AAV2 library was constructed with saturation mutagenesis at the capsid site 548. A total of 10^12^ v.g. of this library was intravenously injected into SCID mice pretreated with a physiological concentration of human IVIG 24 hours prior. Mouse livers were collected for genomic DNA extraction and capsid gene sequencing 2 weeks later. (B) Characterization of AAV2 variants on their tissue tropism in the mouse liver. A total of 10^10^ v.g. of pooled AAV2 variants of the same ratio was injected intravenously into the SCID mice without IVIG pretreatment. Mouse livers were collected for examination of gene frequencies of AAV2 capsid genes 2 weeks later. (C) Neutralization effects on AAV2 variants by individual human plasma samples exemplified by the plasma sample number 40#. AAV2 variants harnessing the lacZ reporter gene were incubated with serially diluted human plasma samples for 1 hour at 37°C before inoculation into 293 cells. The cells were harvested for the β-galactosidase assay 24 hours later. (D) Neutralization effects of human IVIG on AAV2 variants. The results were collected from four mice in (B) and from two cell monolayers in (C) and (D). One-way ANOVA followed by Dunnett’s multiple comparisons test was performed to compare the other AAV2 variants to AAV2-E548 in (B), (C) and (D), and significant differences at the 0.05 level are indicated by asterisks. The data are shown as the mean values ± SDs.

After one round of screening in SCID-IVIG mice, nine types of AAV2 variants were retrieved from the mouse liver by genomic DNA extraction and PCR, and their gene frequencies are shown in Fig 7A. Interestingly, neither G548 nor E548 was dominant among the AAV2 variants. The gene frequencies of the AAV2 variants ranged from high to low: V548 (37.7%), C548 (18.9%), M548 (13.2%), E548 (11.3%), I548 (9.4%) and G548 (1.9%). AAV2 variants retrieved from mouse liver were infused into SCID-IVIG mice for further screening. Unexpectedly, none of the AAV2 variants survived this screening. These results suggested that AAV2 variants with mutations at a single site could not survive the rigorous selection pressure from pooled human plasma, possibly due to the high prevalence of AAV2-neutralizing antibodies in humans [37] as well as the neutralization epitopes globally distributed over the exterior surface of AAV capsids [42].

Although it was verified that mutation at a single site could not significantly alter the immunogenicity of the AAV capsid, we were interested in characterizing the AAV2 variants from the first round of screening. AAV2 vectors containing their own capsid genes were mixed at the same ratio before being administered to SCID mice to investigate their tissue tropism. Specifically, immunological selection pressure was excluded from this characterization without IVIG treatment during the experiment. Intriguingly, the transduction efficiency of AAV2-E548 in the SCID mouse liver was approximately 15-fold greater than that of AAV2-G548 (49.6% versus 3.3% in gene frequency) and was significantly greater than those of any of the other AAV2 variants (p < 0.05, Fig 7B). To further dissect the evolutionary significance of these results, the transduction efficiencies of AAV2-G548 and E548 were compared in the human hepatoma Huh7 cell line. Interestingly, AAV2-E548 transduced these human cells only slightly more efficiently than did AAV2-G548 (145,340 versus 126,518 RLU /mg protein at an MOI of 1500, p = 0.01, S2 Fig). This observation implied that the selective advantage of AAV2-E548 over AAV2-G548 in the transduction of mouse liver tissue might not completely reflect their tissue tropism in humans, as AAV2 and AAV8 previously exhibited similarly variable performance in mouse and human hepatocytes [43].

In addition to tissue transduction, the mutational effects of the 548 site in AAV2 variants were further examined for neutralization by individual and pooled human plasmas. The four human plasma samples with significant variation in binding and neutralization of AAV2-E548 and G548 (Fig 6B–6D) exhibited similarly variable neutralization effects on the six AAV2 variants, with AAV2-E548 always showing the highest IC_50_, G548 and C548 showing the median, and M548, V548 and I548 showing the lowest. With human plasma sample number 40# as an example, AAV2-E548 showed an IC_50_ value approximately twofold greater than of that of AAV2-G548 (0.0188 versus 0.0098 in plasma dilutions), with significantly reduced neutralization sensitivity over all the other five AAV2 variants (p < 0.05, Fig 7C). When IVIG was used for the neutralization assays, a similar variation pattern of neutralization effects was observed for the six AAV2 variants, which could be divided into three categories (Fig 7D). Although the magnitude of variation among AAV variants was not as large as that for individual human plasma samples, AAV2-E548 was still significantly less sensitive to neutralization by IVIG than any of the other five AAV2 capsids when IC_50_ values were calculated for comparison (p < 0.05, Fig 7D). These results further validated the selective advantage of AAV2-E548 by human neutralizing antibodies in comparison to other AAV2 variants at both the individual and population levels.

## Discussion

Homologous recombination has been shown to play an important role in the genetic diversity of human and nonhuman primate AAVs [4,23]. This study provided further insights into the evolutionary mechanism of AAVs, with a focus on the role of positive selection in the diversification of their lineages.

Two representative programs, CODEML [22] and MEME [18], were utilized for the detection of positive selection during the evolution of AAV lineages. As a codon-based algorithm, the M8 model in CODEML was efficient for detecting pervasive positive selection, such as the T410 site in the AAV2 lineage (Table 1), which reflected the overlaid evolutionary events across the different AAV lineages, including T410 in the AAV2 lineage, T411 in the AAV1/AAV6 lineage, Q413 in the AAV8 lineage and Q412 in the AAV9 lineage, all of which were detected by the MEME program (Table 2). Similarly, the selected site R710 was detected by MEME as an earlier evolutionary event in the AAV5 branch (Table 2), which corresponded to the S721 site in the AAV2 capsid gene inferred from the M8 model (Table 1). Thus, both CODEML and MEME were useful for detecting positive selection in this study, but MEME tended to be more useful for detecting sporadic diversifying selection dispersed among the different AAV lineages. Furthermore, the interference of recombination might need to be removed before positive selection is detected by spitting the protein coding sequences via recombination breakpoints [24,25]. However, no positive selection was detected by the M8 model after the removal of recombination (Table 1), while numerous selected sites were effectively identified by the MEME program (Table 2). These results suggested that MEME might be more robust for the detection of positive selection when recombination effects need to be removed via the sequence-splitting method.

Various biological models, including human PBMCs and BALB/c mice/adenoviruses for cellular immunity (Fig 4), mouse monoclonal antibodies C11 and A20 (Fig 5), individual and pooled human plasmas for humoral immunity (Fig 6), and SCID mice/IVIG (Fig 7) and human Huh7 cells (S2 Fig) for directed evolution, have been used to dissect the immunological drives of AAV2 evolution. Considering these combined data, it was concluded that selective pressure from human neutralizing antibodies might play a crucial role in the accelerated evolution of the AAV2 capsid site 548, in addition to its relatively minor role in host adaptation. Furthermore, this observation was mostly substantiated by the results from human neutralizing antibodies and cell lines (Figs 6 and S2), which emphasized the cautious selection of biological models for the experimental validation of the evolutionary hypotheses of AAV [43].

One key finding in this study was that the AAV2-G548 to E548 replacement was positively selected by human neutralizing antibodies. Although AAV2-E548 was obviously more prevalent and high titers of its binding antibodies were detected in the human population (Fig 6A and 6E), it was less neutralized by human neutralizing antibodies than were the other AAV2 variants at both the individual (Fig 6B–6D) and population (Fig 6F) levels. Neutralizing antibody evasion has been extensively investigated in studies of human pathogens such as HIV-1 [12] and HCV [13]. Among these RNA viruses, it was clear that the contemporaneous viruses were continuously less susceptible to neutralization than were the viruses sampled earlier in the same human subject. Furthermore, the neutralizing titers of the human plasma samples for the autologous viruses were significantly greater than those for the heterologous viruses. Some important evolutionary implications could be made through the comparison of these results. As RNA viruses, HIV-1 and HCV have evolved at high rates because of the low fidelity of their RNA polymerases. The rapid evolution of their genomes was coupled with positive selection and immune evasion at the individual level in only months or years, which correlated with the fact that human plasma samples can neutralize autologous early viruses, but not autologous late viruses or heterologous viruses with significant variations [12,13].

As AAV is a ssDNA virus, the genome has evolved at a relatively slower rate because of the fidelity of the host DNA polymerase. In accordance with this, mutations and the selection of AAV variants might occur at the population level on a large time scale (likely hundreds or thousands of years, which was inferred by the similar distributions of AAV serotypes in human populations from both the United States [5,6] and China [44]). With a selective advantage in immune evasion, AAV2-E548 could be spread throughout whole human populations and maintained for many generations. Therefore, AAV2-E548 was more prevalent than the ancestral AAV2-G548, which was inferred from its higher antibody titers in most human plasma samples (Fig 6A). Furthermore, it tended to be less neutralized by most of the individual human plasma samples as well as the pooled human plasma (Fig 6B–6F). Overall these data suggested that RNA and DNA viruses might share analogous evolutionary mechanisms for neutralization evasion but behave much differently in their adaptive patterns in terms of time scale and spatial range, which is genetically determined.

It will be interesting to further elucidate the structural mechanism underlying the immune evasion of AAV2 by mutations at site 548. In previous work, McCraw et al. [33] determined the structure of the AAV2 capsid-mouse A20 monoclonal antibody complex (S3 Fig). E548 was found to be included in the contact region between AAV2 and the A20 antibody but was not critical for this interaction. In accordance with this information, the E548G replacement significantly reduced the binding of the AAV2 capsid to the A20 antibody but did not markedly influence the neutralization effects (Fig 5D and 5E). Furthermore, mutational effects on antibody binding and neutralization were evaluated for numerous sites on the external surface of the AAV2 capsid including E548 and V708, which are both involved in interactions of the AAV2-A20 complex [45]. In comparison to the weaker contact of E548 with the AAV2 capsid, V708 was potentially more important for the AAV2-A20 interaction [33]. Correspondingly, the V708K mutation not only attenuated A20 binding by 10-fold but also reduced A20 neutralization by 220-fold. Interestingly, the performance of V708K and E548G in interactions with human antibodies was the exact opposite [45]. The V708K mutant was not resistant to human IVIG neutralization, but the E548G replacement resulted in a significant alteration in the neutralization of human antibodies (Fig 6). These results suggested that the 548 site of the AAV2 capsid might be critical for interaction with human antibodies, while the 708 site might not be significant for this process. This hypothesis could be further tested in cases in which human neutralizing antibodies were isolated and structurally characterized against the AAV2 capsid, especially from human patients receiving gene therapy [15].

Rapid evolution was deduced to be associated with the emergence of carnivore parvovirus [46]. Although not examined in this study, positive selection might play an important role in shaping the diverse tissue tropisms of AAV lineages, with two selected sites, R710 and Q594, identified from the AAV5 and AAV8 capsid genes, respectively, potentially interacting with AAVR (Tables 2 and 3 and Fig 1). Intriguingly, AAV5 is the AAV with the most distant evolutionary relationship with other AAV serotypes [47], and AAV8 represents an AAV clade that is prevalent in both human and nonhuman primates [5]. Further work on the construction of AAV mutants at these sites and evaluation of their effects on tissue tropism will help to elucidate the role of positive selection in the cross-species transmission of AAV lineages.

AAV has been largely adopted as a gene therapy vector, even with pre-existing human neutralizing antibodies being one of the important obstacles in its application [15]. In this study, experimental evolution was implemented to mutate and select AAV2 (at the 548 site) and AAV8 (at the 551 and 594 sites) for more efficient evasion of human neutralizing antibodies (Fig 7). The AAV2 variants disappeared after two rounds of screening in the SCID-IVIG mice. The AAV8 variants were completely reversed to the wild-type status after one round of screening, which suggested the strong functional constraints on these two selected sites. These data suggested that it might not be feasible to engineer an AAV vector with novel immunogenicity with only a few mutations because of functional constraints as well as the existence of human neutralizing antibodies with neutralization epitopes dispersed over the exterior surface of AAV capsids [48]. Many more variations might need to be introduced into the AAV capsid without destabilizing its structural stability to achieve this goal. Moreover, closely monitoring the evolution of circulating AAVs in human subjects in clinical trials before and after treatment might be particularly interesting [15,48]. Our research suggested that neutralization evasion might be a relatively slow and effective adaptation process for wild-type AAVs during their natural evolution. However, very strong humoral immunity could be elicited against AAV capsids in human subjects because of the administration of a high vector dose in a short time. Accelerated evolution of AAV serotypes could occur in these patients, which might increase safety concerns but could also favor the isolation of novel AAV variants with the potential immune evasion of human neutralizing antibodies from biopsies [44].

This study, taken in its entirety, demonstrated a trend for positive selection to play a role in the evolution of human and nonhuman primate AAVs. Furthermore, immune escape was revealed as one of the major driving forces for the diversification of AAV lineages. Our study provides a foundation for further elucidation of the evolutionary mechanism of AAV serotypes and their genetic engineering, which may ultimately contribute to their application in human gene therapy.

## Materials and methods

### Ethics statement

The Biomedical Ethical Committee of the West China Hospital of Sichuan University approved the collection of patient blood samples in this study with approval No. 2014(82). The patients provided a written informed consent before the procedure after receiving a thorough explanation of the purposes of the research. Patient records were anonymized and de-identified prior to analysis.

### Sequence retrieval and alignment

The 146 complete coding sequences of human or nonhuman primate AAV capsid genes were downloaded from GenBank in May 2012 (http://www.ncbi.nlm.nih.gov/). After removal of the 16 artificial mutants and rh.23 with a large sequence deletion, 129 AAV sequences were aligned using ClustalX2.1 [49] (information on these sequences is listed in S1 Table). The resulting fasta file was further re-aligned using the codon algorithm implemented in PRANKSTER [50] to improve the quality of the alignment [19,20].

### Detection and elimination of recombination

Likelihood test often mistakes recombination as evidence for positive selection when the recombination level is high [24]. The robust inference of positive selection can be achieved by allowing tree topologies and branch lengths to change across detected recombination breakpoints [25]. GENECONV [51] and MaxChi [52] are two substitution methods with greater power and accuracy in the detection of recombination [53,54] (Martin DP, personal communication). GARD is another useful phylogenetic method for the detection of recombination [27]. These three programs were simultaneously used in the detection of recombination breakpoints among AAV capsid gene sequences to attain maximum power. The AAV sequence alignment was partitioned into 23 larger segments that contained no recombination breakpoints with codon numbers greater than 3. Tree topologies were inferred from these sequence segments by the maximum likelihood method implemented in DNAML of PHYLIP3.69 [21].

### Positive selection through the AAV lineages

The CODEML program of PAML4.6 [22] is popular for the detection of positive selection across multiple paralogous gene lineages. The nested evolution models M1a/M2a and M7/M8 were compared using likelihood ratio tests to assess the significance of positive selection, and empirical Bayes methods allowed the identification of individual positively selected codon sites a posteriori. The results from the Bayes empirical Bayes analysis were adopted as recommended by the software developer.

### Episodic diversifying selection dispersed among AAV lineages

The assumption in site models that selective pressure remains constant across lineages might be unrealistic. The branch-site test implemented in CODEML [17] and the mixed effects model of evolution implemented in MEME [18] allow selection pressure to vary both among sites in the protein and across branches on the tree. These approaches were then performed for the detection of episodic diversifying selection among AAV lineages. With respect to the branch-site test, a foreground branch was required to be assigned a priori. The five AAV lineages represented by AAV2, AAV1/AAV6, AAV5, AAV8 and AAV9 were selected for the test because of their distinct biological properties and important applications in gene therapy. The status of the foreground branch was determined in the tree topology because it could form an independent AAV clade and is closely related to the emergence of a specific AAV serotype. The unrecombined AAV sequence segments and their corresponding tree topologies were then sequentially subjected to a branch-site test, and the positively selected sites from Bayes empirical Bayes analysis were adopted. The unrecombined AAV sequence segments were directly used for MEME analysis without providing tree topology or designation of the foreground branch a priori. However, the positively selected branches had to be visually identified after detection of the codons subject to diversifying selection. For both of these methods, identical serine residues were observed throughout all the AAV lineages but were detected as positively selected by the program, which was thought to represent a case of toggling selection or doublet mutation [29] rather than strict positive selection because no replacement could eventually be observed.

### Localization of positively selected sites in the AAV structure

The positions of the AAV amino acids identified as undergoing accelerated evolution were visualized in the VP3 monomer structures of the respective AAV serotypes using atomic coordinates downloaded from the Protein Data Bank (RCSB PDB codes: 1LP3, 3OAH, 3NTT, 2QA0, and 3UX1 for AAV2, AAV6, AAV5, AAV8, and AAV9, respectively) using the program COOT [55]. Amino acids located within the VP1u or VP1/2 common region of the AAV VPs could not be visualized because of a lack of structural information. A review of the literature was performed for functional annotation of the amino acids within VP3. The coordinates for the 60-mer capsids of each AAV were generated from the VP monomer structures by icosahedral matrix multiplication at the VIPERdb website (viperdb.scripps.edu), and the results were then employed to generate images in the PyMol program to visualize the surface juxtaposition of the amino acids [56]. Two-dimensional “Roadmap” images were also generated from the RIVEM program for a more detailed visualization of the positions of these residues on the capsids [57].

### Enzyme-linked immunosorbent assays (ELISAs) against AAV capsids

ELISA was employed to examine the existence of the AAVhu.1 antibody response in individual human plasma samples and the binding of AAV2 variants to mouse antibodies, as shown in Fig 5, and individual and pooled human plasmas, as shown in Figs 6 and 7. First, 96-well microtiter plates were coated with 10^9^ vector genomes of AAV variants in 0.1 M sodium bicarbonate buffer, pH 9.6, overnight at 4°C. The plates were blocked with phosphate-buffered saline (PBS), 3% bovine serum albumin (BSA), and 0.05% Tween 20 at room temperature for 2 hours and then incubated with antibodies serially diluted in PBS containing 1% BSA at 37°C for 1.5 hours. Unbound antibodies were removed by washing with three aliquots of PBS containing 0.05% Tween-20. Then, 1:5,000 diluted goat anti-human (Sigma) or anti-mouse IgG (Boster) coupled to horseradish peroxidase was added for incubation at 37°C for 1.5 hours. After washing three times with PBS containing 0.1% Tween-20, 100 μl of tetramethylbenzidine (TMB) substrate (Thermo) was added and incubated at room temperature for 15 minutes. Then, 50 μl of 2 M sulfuric acid was added to stop the reaction, and the optical density was measured at an absorbance wavelength of 450-nm using a Multiskan MK3 plate reader. The OD values versus antibody dilution ratios or concentrations were plotted to fit a sigmoidal curve in Origin7.5. The antibody binding capacity was determined as the half-maximal effective concentration (EC_50_).

### Isolation of human peripheral blood mononuclear cells (PBMCs) and ELISpot assays for the production of IFN-γ

A cohort of 94 gastric cancer patients was recruited from West China Hospital, Sichuan University, for the study of the immune response to AAV2 variants. The AAV hu.1 capsid gene (GenBank No AY530575) was synthesized for packaging of recombinant virus as an antigen for ELISA screening of human plasma samples because its capsid harnessed the peptide 82 sequence [15], which was very similar to that of wild-type AAV2, with only one replacement at the 410 site from T to Q, which was completely identical to that of the AAV2-Q410 capsid. Considering its high prevalence in human populations [5], the AAVhu.1 capsid serves as a valuable agent for the identification of individuals with potential cellular immune responses to the peptide 82-Q410. After ELISAs using AAVhu.1 capsids, whole blood samples from human subjects with positive antibody responses with IVIG as a control were used for the isolation of PBMC using Histopaque-1077 reagent (Sigma) according to the manufacturer’s instructions. ELISpot was then used to further identify individuals with active IFN-γ secretion after stimulation with the peptide 82-Q410 via the Human IFN-γ ELISpot Development Module and Color Module (R&D Systems). A total of 10^6^ of human PBMCs were incubated with 2 μg/ml of peptide 82-Q410 (GNNFQFSYTFEDVPF) at the final concentration for 24 hours before INF-γ secretion was evaluated. Phytohemagglutinin (PHA; Sigma) at a final concentration of 10 μg/mL and media alone were used as the positive and negative controls, respectively. Assays were scored as positive if the number of spots was greater than 3× the media control and > 10 spots per 3× 10^5^ PBMCs. PBMCs with positive scores were further compared for their potential to secret IFN-γ after stimulation with peptide 82-Q410 or peptide 82-T410 (GNNFTFSYTFEDVPF) as described above.

### Construction of recombinant adenovirus and detection of the T-cell response against AAV2 capsids in mice

E1/E3-deleted adenovirus type 5 expressing the wild-type AAV-2 VP1 capsid protein was generated with an AdMax adenovirus vector creation kit (Microbix Biosystems). Briefly, the wild-type AAV-2 capsid gene was amplified by PCR and inserted into a pDC316 donor plasmid. The donor plasmid expression cassette, composed of the cytomegalovirus (CMV) promoter and the wild-type AAV2 capsid gene (AAV2-E548), was recombined into an acceptor vector containing adenoviral genes through Cre-LoxP recombination when co-transfected into HEK-293 cells. After two weeks of culture, when the first signs of cytopathic effects were evident, the cells were harvested, and the lysate was used for plating on HEK-293 cells for plaque purification. After sequence determination of the AAV2 capsid gene, the recombinant Ad5 was used for amplification and large-scale preparation on 30 15-cm plates of HEK-293 cells. Adenovirus purification was performed by two rounds of cesium chloride gradient centrifugation. The viral titer was estimated by determining the OD at 260 nm after denaturation of the virus in 0.1% SDS. The virus was kept at −80°C in buffer containing 50% glycerol and dialyzed in PBS to remove any residual cesium chloride before use in animals. Four mice were immunized intramuscularly with 10^11^ vector genomes of the Ad5-AAV2Cap in 100 μl of PBS. Nine days after immunization, the spleens were harvested from the immunized or naive control mice into 1× Liebowitz’s-15 (L-15) medium (Corning). The spleens were homogenized and filtered through a 70-μm cell strainer. The cells were centrifuged for 10 min at 1200 rpm at 24°C and then incubated with red cell lysis buffer (Gibco) for 5 min, followed by two washes with 2-MLC medium (DMEM, 2% FBS, 1% Pen–Strep, 10 mM HEPES, 1 mM sodium pyruvate, 0.1 mM nonessential amino acids, 1 μM 2-mercaptoethanol). The cells were resuspended in 2-MLC medium at a concentration of 10^7^ cells/ml. The cells were then added to a round-bottom 96-well plate (10^6^/well in triplicate) for intracellular cytokine staining. Peptide 82-Q410 or peptide 82-T410 was added to the cells at a final concentration of 5 μg/ml. PMA/ionomycin (Sigma) at final concentrations of 0.05 and 1 μg/ml, respectively, was used as a positive control. Golgi Stop (BD Pharmingen) was added to each well according to the manufacturer’s instructions. After incubation for 5 hours in 10% CO_2_ at 37°C, the cells were stained with a FITC-conjugated anti-CD4 antibody (BD Pharmingen) for 30 min at 4°C. The cells were washed two times with PBS containing 1% FBS before being resuspended in Cytofix/Cytoperm (BD Pharmingen) for 20 minutes. The cells were washed two times and resuspended in 1× Perm Wash (BD Pharmingen) for 15 minutes at 4°C. The cells were then stained with PE-conjugated anti-IFN-γ diluted in Perm Wash for 30 minutes at 4°C. The cells were washed two times with Perm Wash and one time with PBS and resuspended in PBS for flow cytometry analysis using a BD LSRFortessa (BD Biosciences).

### Preparation of mouse monoclonal antibodies against AAV2 capsids

Three AAV vectors were produced by co-transfection of HEK-293 cells using AAV packaging plasmids together with an adenovirus helper, followed by purification by CsCl ultracentrifugation [58]. The AAV2-G548 capsid gene was modified from AAV2-E548 by PCR mutagenesis and used for the production of a recombinant AAV encompassing the lacZ reporter gene. The AAV2.1 and AAV1.2 capsid genes were synthesized for the production of wild-type AAVs based on comparisons of the AAV2 and AAV1 capsid sequences, respectively, with the A20 antibody binding footprint as a reference [33]. AAV2.1 was engineered based on the AAV2 scaffold with its A20 contact residues removed, and AAV1.2 was engineered based on the AAV1 scaffold with replacements introduced for the plausible binding of the A20 antibody (S1 Fig). Female BALB/c mice were intramuscularly injected with 25 μg of recombinant AAV2-G548 vector together with 50 μl of QuickAntibody Adjuvant (Biodragon). After two boosts in the following 5 weeks, the mice were sacrificed, and their splenocytes were collected for fusion with SP2/0 cells at a ratio of 5:1. The hybridoma cells were cultured in microplates in RPMI 1640/HAT medium after limited dilution. The culture supernatants were first collected for ELISA screening with effective AAV2-G548 capsid binding. Hybridoma clones positive in this screening were further negatively screened for AAV2.1 capsid binding with the A20 contact residues removed and then positively screened for AAV1.2 capsid binding with the A20 interaction regions introduced (Fig 5A). The hybridoma clones surviving the three rounds of screening were subjected to western blotting to validate the interaction of their secreted antibodies with the AAV2-G548 capsid. The hybridoma clones with the highest antibody production were injected into the abdominal cavities of BALB/c mice for ascites collection and IgG purification by affinity chromatography using a HiTrap rProtein A FF column (Cytiva).

### Neutralization antibody assay against AAV2 variants

An *in vitro* neutralization assay was performed to evaluate the immune evasion of AAV2 variants against mouse monoclonal antibodies (Fig 5), individual human plasma samples and human intravenous immunoglobulin (IVIG) (Figs 6 and 7). HEK-293 cells were passaged into 24-well plates at a 1:3 ratio 24 hours before the experiment. Test antibodies were serially diluted 2-fold in naive mouse serum. AAV2 variants harnessing the LacZ reporter gene were diluted to 2 x 10^11^ vector genomes/ml in DMEM. Equal volumes (22 μl) of virus and antibody were mixed and incubated at 37°C for 1 hour. Forty microliters of each reaction mixture was added to 460 μl of DMEM supplemented with 10% FBS, and the mixture was added to two wells of HEK-293 cells. Two controls were used, including naive mouse serum incubated with virus and no transduction. After 1.5 hours at 37°C, the wells were washed once with DMEM, and 500 μl of DMEM supplemented with 10% FBS was added. After another 24 hours at 37°C, the cells were collected and lysed for β-galactosidase assays using the Galacto-Light Plus System (Thermo). The percent neutralization of transduction versus antibody dilution was plotted to fit a sigmoidal curve in Origin7.5. The neutralizing titer was determined as the antibody dilution at which 50% or greater inhibition occurred (IC_50_).

### Engineering of AAV capsid genes and their screening in mice infused with IVIG

The selected sites of the AAV2 and AAV8 capsid genes on the external surfaces of the virions (Fig 1A and 1D) were mutated and screened for AAV variants with more effective evasion of human neutralizing antibodies. Saturation mutagenesis was performed at the E548 site of the AAV2 capsid gene and the A551 and Q594 sites of the AAV8 capsid gene using the QuikChange Lightning Multi Site-Directed Mutagenesis Kit (Agilent). The engineered capsid genes were subcloned into the UF1-AAV8 vector containing the AAV2 ITR and rep gene via HindIII/NotI digestion. The resulting plasmid libraries were utilized for the production of AAV2 and AAV8 saturation mutagenesis libraries with the AAV capsids each packaging their own genome according to Muller et al. [59]. Twenty-four hours later, male 7-week-old CB17 SCID mice (Vital River) were infused with 291 μl of 50 mg/ml IVIG (Shuanglin Biopharmaceutics) to an approximate physiological level of 5 mg/mL, similar to that in the normal human body [41]. A total of 10^12^ vector genomes of AAV2 and 10^11^ vector genomes of AAV8 libraries were injected into two groups of mice via the tail vein. Two weeks later, mouse livers were collected for genomic DNA extraction and PCR retrieval of AAV capsid genes. The PCR products were digested with HindIII/NotI and subcloned into the UF1-AAV8 vector. Tens of random colonies were picked for sequencing of the capsid genes for the AAV2 and AAV8 libraries. Because genetic variations were only identified for the AAV2 capsid gene, its derived AAV library was subjected to a second round of screening in SCID mice after IVIG infusion.

### Characterization of tissue tropism of the AAV2 variants

The fitness of the AAV2 mutants screened in SCID/IVIG mice was further investigated in an *in vivo* mouse model and in a human cell line. To characterize their tissue distribution in mice, packaging plasmids containing the six AAV2 capsid mutants were mixed in the same ratio for the transfection of HEK293 cells and the production of an AAV2 mutant library. A total of 10^10^ vector genomes of this library were injected into four male 7-week-old CB17 SCID mice (Vital River) via the tail vein without antibody infusion. Two weeks later, the mouse livers were collected for genomic DNA extraction, PCR amplification, cloning and sequencing of the AAV2 capsid genes. Approximately 30 gene variants were sequenced for each mouse to calculate the gene frequencies of the AAV2 mutants. With respect to their characterization in humans, recombinant AAV2 vectors containing the lacZ reporter gene were inoculated into human hepatocellular carcinoma Huh7 cells seeded in 24-well plates 24 hours earlier at the indicated infection multiplicities (S2 Fig). After another 48 hours, the cells were lysed for the β-galactosidase assay as described above.

## Supporting information

S1 Fig Amino acid sequences of the AAV capsids used for screening of mouse monoclonal antibodies. The sequences were aligned using the ClustalX2.1 program [49]. The contact residues of AAV2-A20 antibody complex are colored in red in the AAV2 capsid sequence [33]. Their orthologous residues are colored in blue in the AAV1 capsid sequence. The contact residues variable between AAV2 and AAV1 capsid sequences are underlined. To construct AAV2.1, the variable residues in AAV2 capsid were replaced with the corresponding ones from AAV1 capsid (blue-marked). To construct AAV1.2, the variable residues in AAV1 capsid were replaced with the corresponding ones from AAV2 capsid (red-marked), except that the G549 was retained to be in consistent with that of AAV2-G548.
(TIF)

S2 Fig Comparison of transduction efficiencies of two AAV2 variants in the human hepatocytes. AAV2-G548 and AAV2-E548 recombinant vectors encompassing lacZ reporter genes were inoculated into the Huh7 cells at the indicated multiplicity of infection (MOI). Cells were lysed for β-galactosidase assay 48 hours later. The indicated p-values were obtained from paired t-tests. The data are shown as the mean values ± SDs.
(TIF)

S3 Fig Structural insights into the neutralization mechanisms of two AAV2 variants by the mouse antibody A20. (A, B) The 3D structure of AAV2-E548 bound to the A20 antibody (PDB ID: 3JIS) [33]. (C, D) The putative structure of AAV2-G548 bound to the A20. The AAV2-G548 structure was predicted using AlphaFold [60] and aligned with the structure of AAV2-A20 complex (PDB ID: 3JIS) in PyMOL interface. The AAV2 capsid is shown in surface model and its contact residues with A20 are colored in red for E548 or G548, in green for V708, and in orange for the rest. The A20 antibody is shown in ribbon representation composed of the heavy chain (cyan) and the light chain (magentas).
(TIF)

S1 Table List of 129 AAV capsid gene sequences used for selection analysis. (DOC)

S2 Table MHCII peptides identified from AAV2 VP1 capsid sequence. (DOC)
