# Supplementary figures and images for: Dissecting positive selection events and immunological drives during the evolution of adeno-associated virus lineages
Source: PLoS Pathog. 2024 Jun 17;20(6):e1012260. doi: 10.1371/journal.ppat.1012260 (PMC11182496; doi:10.1371/journal.ppat.1012260)

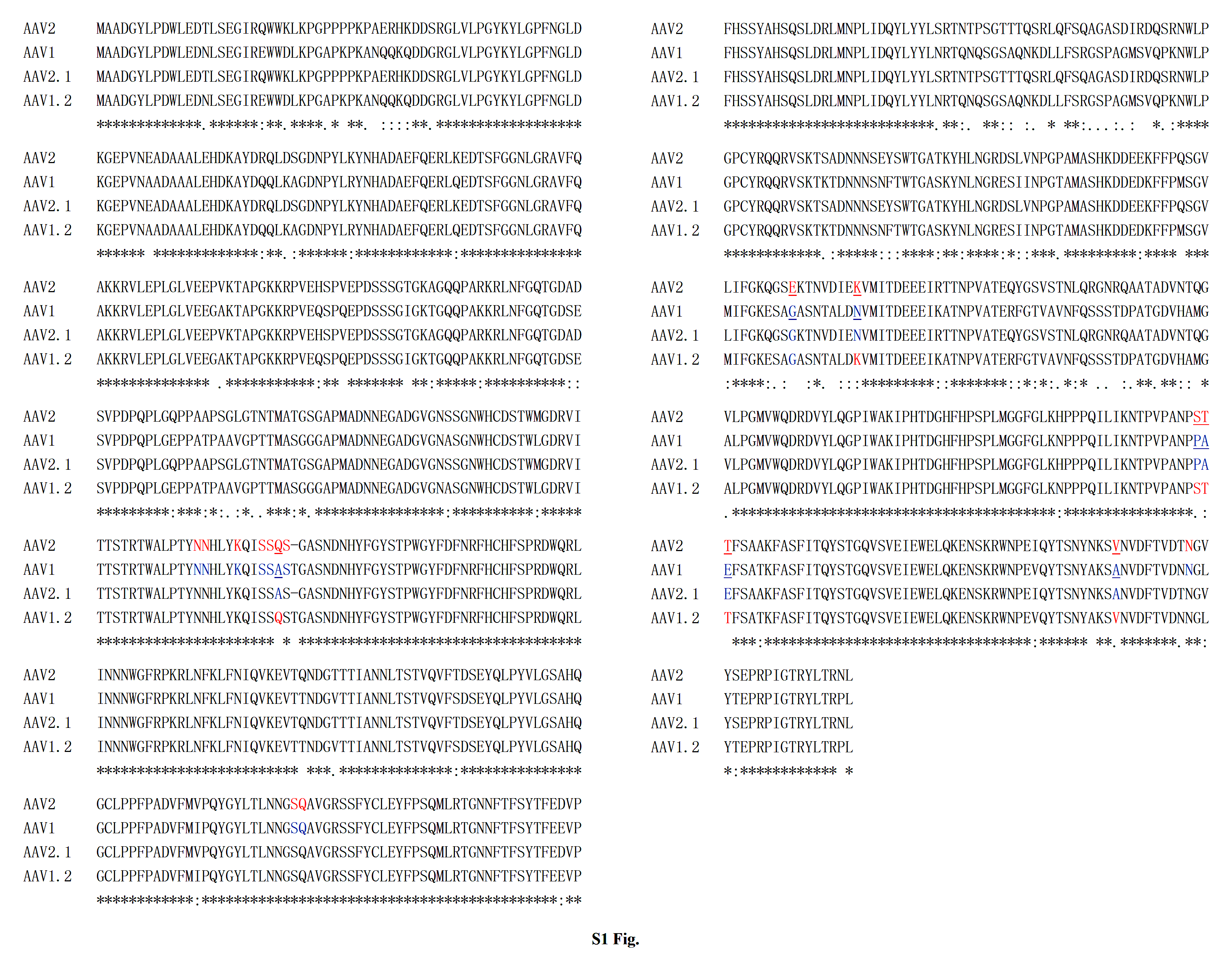

Supplement: S1 Fig — The sequences were aligned using the ClustalX2.1 program [49]. The contact residues of AAV2-A20 antibody complex are colored in red in the AAV2 capsid sequence [33]. Their orthologous residues are colored in blue in the AAV1 capsid sequence. The contact residues variable between AAV2 and AAV1 capsid sequences are underlined. To construct AAV2.1, the variable residues in AAV2 capsid were replaced with the corresponding ones from AAV1 capsid (blue-marked). To construct AAV1.2, the variable residues in AAV1 capsid were replaced with the corresponding ones from AAV2 capsid (red-marked), except that the G549 was retained to be in consistent with that of AAV2-G548. (TIF) [file ppat.1012260.s001.tif]

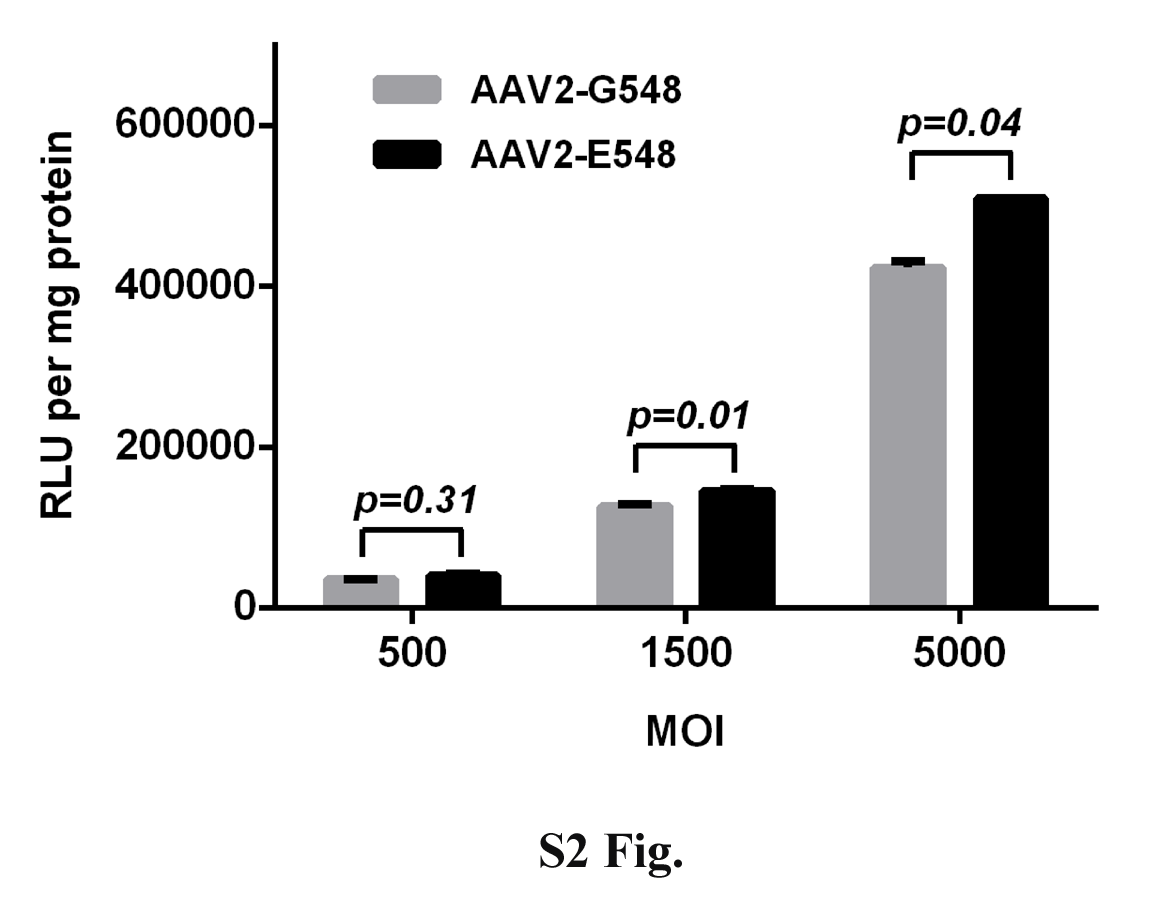

Supplement: S2 Fig — AAV2-G548 and AAV2-E548 recombinant vectors encompassing lacZ reporter genes were inoculated into the Huh7 cells at the indicated multiplicity of infection (MOI). Cells were lysed for β-galactosidase assay 48 hours later. The indicated p-values were obtained from paired t-tests. The data are shown as the mean values ± SDs. (TIF) [file ppat.1012260.s002.tif]

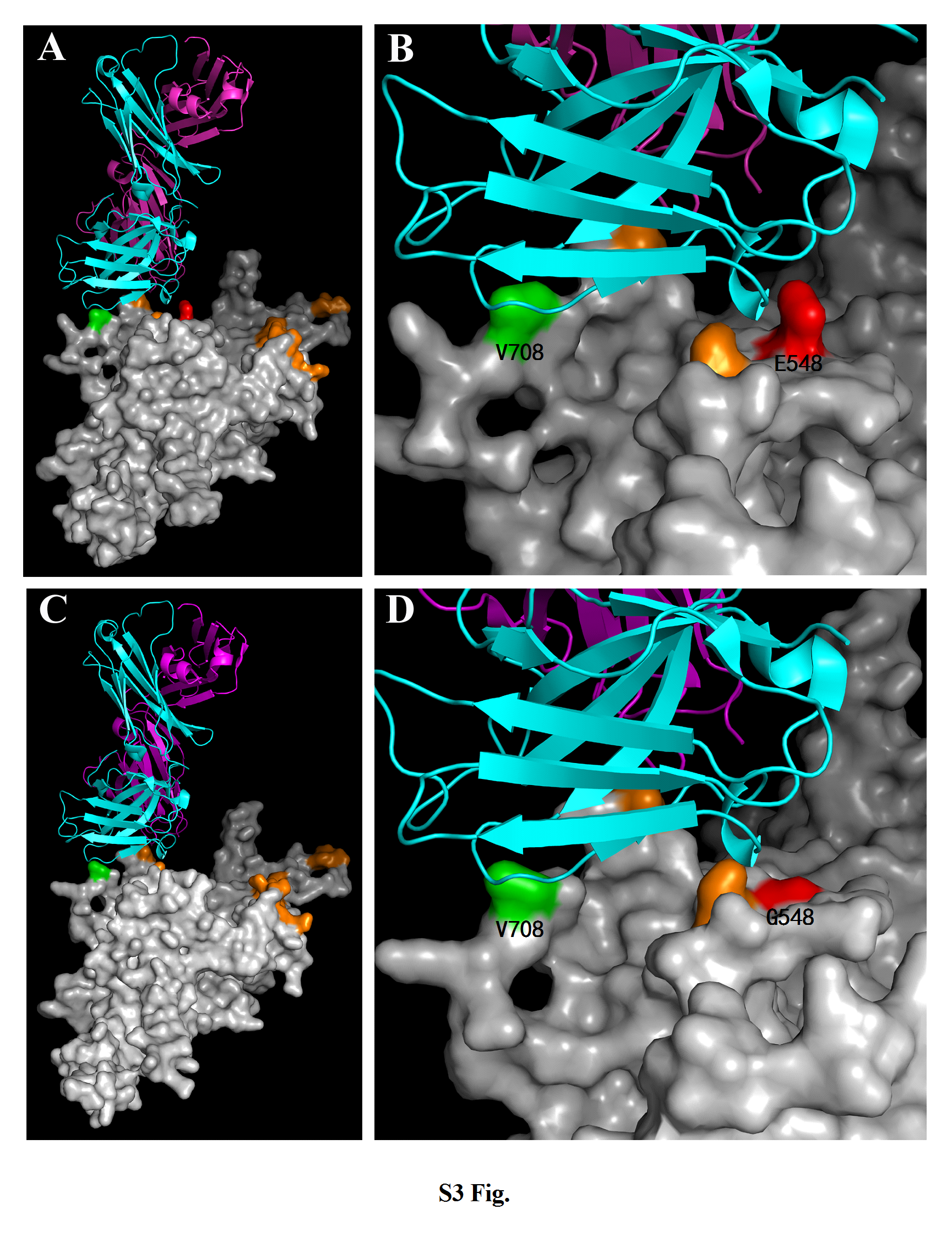

Supplement: S3 Fig — (A, B) The 3D structure of AAV2-E548 bound to the A20 antibody (PDB ID: 3JIS) [33]. (C, D) The putative structure of AAV2-G548 bound to the A20. The AAV2-G548 structure was predicted using AlphaFold [60] and aligned with the structure of AAV2-A20 complex (PDB ID: 3JIS) in PyMOL interface. The AAV2 capsid is shown in surface model and its contact residues with A20 are colored in red for E548 or G548, in green for V708, and in orange for the rest. The A20 antibody is shown in ribbon representation composed of the heavy chain (cyan) and the light chain (magentas). (TIF) [file ppat.1012260.s003.tif]
