# Supplementary material for: Dissecting positive selection events and immunological drives during the evolution of adeno-associated virus lineages
Source: PLoS Pathog. 2024 Jun 17;20(6):e1012260. doi: 10.1371/journal.ppat.1012260 (PMC11182496; doi:10.1371/journal.ppat.1012260)
Supplement: S1 Table — (DOC) [file ppat.1012260.s004.doc]

**S1 Table**. List of 129 AAV capsid gene sequences used for selection analysis.

| AAV variant | GenBank Accession No. | Origin |
| --- | --- | --- |
| AAV1 | AF063497 | Rhesus macaque |
| AAV2 | AF043303 | Human |
| AAV3 | NC_001729 | Human |
| AAV3B | AF028705 | Human |
| AAV4 | NC_001829 | Green monkey |
| AAV5 | NC_006152 | Human |
| AAV6 | AF028704 | Human |
| AAV7 | NC_006260 | Rhesus macaque |
| AAV8 | NC_006261 | Rhesus macaque |
| AAV9 | AY530579 | Human |
| AAV10 | AY631965 | Cynomologus macaque |
| AAV11 | AY631966 | Cynomologus macaque |
| AAV12 | DQ813647 | Vervet monkey |
| AAV13 | EU285562 | Vervet monkey |
| bb.1 | AY243023 | Baboon |
| bb.2 | AY243022 | Baboon |
| ch.5 | AY243021 | Chimpanzee |
| cy.2 | AY243020 | Cynomologus macaque |
| cy.3 | AY243019 | Cynomologus macaque |
| cy.4 | AY243018 | Cynomologus macaque |
| cy.5 | AY243017 | Cynomologus macaque |
| cy.6 | AY243016 | Cynomologus macaque |
| hu.1 | AY530575 | Human |
| hu.2 | AY530585 | Human |
| hu.3 | AY530595 | Human |
| hu.4 | AY530602 | Human |
| hu.6 | AY530621 | Human |
| hu.7 | AY530628 | Human |
| hu.9 | AY530629 | Human |
| hu.10 | AY530576 | Human |
| hu.11 | AY530577 | Human |
| hu.13 | AY530578 | Human |
| hu.15 | AY530580 | Human |
| hu.16 | AY530581 | Human |
| hu.17 | AY530582 | Human |
| hu.18 | AY530583 | Human |
| hu.19 | AY530584 | Human |
| hu.20 | AY530586 | Human |
| hu.21 | AY530587 | Human |
| hu.22 | AY530588 | Human |
| hu.23 | AY530589 | Human |
| hu.24 | AY530590 | Human |
| hu.25 | AY530591 | Human |
| hu.27 | AY530592 | Human |
| hu.28 | AY530593 | Human |
| hu.29 | AY530594 | Human |
| hu.31 | AY530596 | Human |
| hu.32 | AY530597 | Human |
| hu.34 | AY530598 | Human |
| hu.35 | AY530599 | Human |
| hu.37 | AY530600 | Human |
| hu.39 | AY530601 | Human |
| hu.40 | AY530603 | Human |
| hu.41 | AY530604 | Human |
| hu.42 | AY530605 | Human |
| hu.43 | AY530606 | Human |
| hu.44 | AY530607 | Human |
| hu.45 | AY530608 | Human |
| hu.46 | AY530609 | Human |
| hu.47 | AY530610 | Human |
| hu.48 | AY530611 | Human |
| hu.49 | AY530612 | Human |
| hu.51 | AY530613 | Human |
| hu.52 | AY530614 | Human |
| hu.53 | AY530615 | Human |
| hu.54 | AY530616 | Human |
| hu.55 | AY530617 | Human |
| hu.56 | AY530618 | Human |
| hu.57 | AY530619 | Human |
| hu.58 | AY530620 | Human |
| hu.60 | AY530622 | Human |
| hu.61 | AY530623 | Human |
| hu.63 | AY530624 | Human |
| hu.64 | AY530625 | Human |
| hu.66 | AY530626 | Human |
| hu.67 | AY530627 | Human |
| hu.LG15 | AY695377 | Human |
| hu.S17 | AY695376 | Human |
| hu.T17 | AY695370 | Human |
| hu.T32 | AY695371 | Human |
| hu.T40 | AY695372 | Human |
| hu.T41 | AY695378 | Human |
| hu.T70 | AY695373 | Human |
| hu.T71 | AY695374 | Human |
| hu.T88 | AY695375 | Human |
| pi.1 | AY530553 | Pig-tailed macaque |
| pi.2 | AY530554 | Pig-tailed macaque |
| pi.3 | AY530555 | Pig-tailed macaque |
| rh.1 | AY530556 | Rhesus macaque |
| rh.2 | AY243007 | Rhesus macaque |
| rh.8 | AY242997 | Rhesus macaque |
| rh.10 | AY243015 | Rhesus macaque |
| rh.12 | AY243014 | Rhesus macaque |
| rh.13 | AY243013 | Rhesus macaque |
| rh.14 | AY243012 | Rhesus macaque |
| rh.16 | AY243011 | Rhesus macaque |
| rh.17 | AY243010 | Rhesus macaque |
| rh.18 | AY243009 | Rhesus macaque |
| rh.19 | AY243008 | Rhesus macaque |
| rh.22 | AY243006 | Rhesus macaque |
| rh.24 | AY243004 | Rhesus macaque |
| rh.25 | AY530557 | Rhesus macaque |
| rh.32 | AY243003 | Rhesus macaque |
| rh.33 | AY243002 | Rhesus macaque |
| rh.34 | AY243001 | Rhesus macaque |
| rh.35 | AY243000 | Rhesus macaque |
| rh.36 | AY242999 | Rhesus macaque |
| rh.37 | AY242998 | Rhesus macaque |
| rh.38 | AY530558 | Rhesus macaque |
| rh.39 | EU368921 | Rhesus macaque |
| rh.40 | AY530559 | Rhesus macaque |
| rh.43 | AY530560 | Rhesus macaque |
| rh.46 | EU368922 | Rhesus macaque |
| rh.48 | AY530561 | Rhesus macaque |
| rh.49 | AY530562 | Rhesus macaque |
| rh.50 | AY530563 | Rhesus macaque |
| rh.51 | AY530564 | Rhesus macaque |
| rh.52 | AY530565 | Rhesus macaque |
| rh.53 | AY530566 | Rhesus macaque |
| rh.54 | AY530567 | Rhesus macaque |
| rh.55 | AY530568 | Rhesus macaque |
| rh.57 | AY530569 | Rhesus macaque |
| rh.58 | AY530570 | Rhesus macaque |
| rh.60 | AY530571 | Rhesus macaque |
| rh.61 | AY530572 | Rhesus macaque |
| rh.62 | AY530573 | Rhesus macaque |
| rh.64 | AY530574 | Rhesus macaque |
| VR-195 | DQ180604 | Cynomolgus macaque |
| VR-355 | DQ180605 | Rhesus macaque |
