# Supplementary material for: Dissecting positive selection events and immunological drives during the evolution of adeno-associated virus lineages
Source: PLoS Pathog. 2024 Jun 17;20(6):e1012260. doi: 10.1371/journal.ppat.1012260 (PMC11182496; doi:10.1371/journal.ppat.1012260)
Supplement: S2 Table — (DOC) [file ppat.1012260.s005.doc]

**S2 Table.** **MHCII peptides identified from AAV2 VP1 capsid sequence.**

| MHCII genotype | Position in AAV2 capsid | Peptide sequence | Optimal score | Binding threshold |
| --- | --- | --- | --- | --- |
| HLA-DR2 | 398-412 | FPSQMLRTGNNF**T**FS | 7.331 | 14.66% |
| HLA-DR4 (DRB1*0401) | 410-424 | **T**FSYTFEDVPFHSSY | 8.688 | 19.71 % |
| HLA-DR5 | 398-412 | FPSQMLRTGNNF**T**FS | 9.746 | 20.05 % |
| HLA-DR8 (DRB1*0801) | 397-411 | YFPSQMLRTGNNF**T**F | 11.875 | 25.13 % |
| I-Ad | 406-420 | GNNF**T**FSYTFEDVPF | 12.157 | 22.88 % |

Note− MHC binding epitopes predicted by RANKPEP with binding threshold greater than 5% were shown. The bolded residues indicate the AAV2 capsid gene 548 site with a high evolutionary rate. The hypothesis that its accelerated evolution was driven by selection pressure from host CD4^+^ T-cells was tested by immunological experiments (Fig 4C and 4D).
